# Sources of variation in baseline gene expression levels from toxicogenomics study control animals across multiple laboratories

**DOI:** 10.1186/1471-2164-9-285

**Published:** 2008-06-12

**Authors:** Michael J Boedigheimer, Russell D Wolfinger, Michael B Bass, Pierre R Bushel, Jeff W Chou, Matthew Cooper, J Christopher Corton, Jennifer Fostel, Susan Hester, Janice S Lee, Fenglong Liu, Jie Liu, Hui-Rong Qian, John Quackenbush, Syril Pettit, Karol L Thompson

**Affiliations:** 1 Amgen Inc., Thousand Oaks, CA 91320, USA; 2 SAS Institute Inc., Cary, NC 27513, USA; 3 NIEHS, Research Triangle Park, NC 27709, USA; 4 Roche Palo Alto LLC, Palo Alto, CA 94304, USA; 5 US EPA, Research Triangle Park, NC 27711, USA; 6 Dana-Farber Cancer Institute, Boston, MA 02115, USA; 7 ICS, NCI at NIEHS, Research Triangle Park, NC 27709, USA; 8 Eli Lilly and Co., Indianapolis, IN 46285, USA; 9 Harvard School of Public Health, Boston, MA 02115, USA; 10 ILSI/HESI, Washington, DC 20005, USA; 11 CDER, US FDA, Silver Spring, MD 20993, USA

## Abstract

**Background:**

The use of gene expression profiling in both clinical and laboratory settings would be enhanced by better characterization of variance due to individual, environmental, and technical factors. Meta-analysis of microarray data from untreated or vehicle-treated animals within the control arm of toxicogenomics studies could yield useful information on baseline fluctuations in gene expression, although control animal data has not been available on a scale and in a form best served for data-mining.

**Results:**

A dataset of control animal microarray expression data was assembled by a working group of the Health and Environmental Sciences Institute's Technical Committee on the Application of Genomics in Mechanism Based Risk Assessment in order to provide a public resource for assessments of variability in baseline gene expression. Data from over 500 Affymetrix microarrays from control rat liver and kidney were collected from 16 different institutions. Thirty-five biological and technical factors were obtained for each animal, describing a wide range of study characteristics, and a subset were evaluated in detail for their contribution to total variability using multivariate statistical and graphical techniques.

**Conclusion:**

The study factors that emerged as key sources of variability included gender, organ section, strain, and fasting state. These and other study factors were identified as key descriptors that should be included in the minimal information about a toxicogenomics study needed for interpretation of results by an independent source. Genes that are the most and least variable, gender-selective, or altered by fasting were also identified and functionally categorized. Better characterization of gene expression variability in control animals will aid in the design of toxicogenomics studies and in the interpretation of their results.

## Background

Animal models are routinely used to assess the risk of exposure to drugs and chemicals for the human population. Whole genome sequencing and microarray technology have added new tools that can be integrated into traditional toxicity testing strategies for enhanced predictive and mechanistic insights. Variations in study design are typical for toxicogenomics studies, but their impact on gene expression in control animals has not been well characterized. Several studies are available that have examined factors contributing to variation in gene expression in human peripheral blood [1,2]. A limited number of studies have been published on individual animal variability [3] and the effect of selected study conditions [4-6] on baseline gene expression patterns in the control arm of toxicity studies in rats.

Databases of historical background levels have utility for toxicologic risk assessment. For example, the Registry of Industrial Toxicology Animal database of historical tumor data is used to interpret tumor incidence rates in long-term rodent carcinogenicity bioassays [7]. In September 2004, the ILSI Health and Environmental Sciences Institute (HESI) Technical Committee on the Application of Genomics in Mechanism Based Risk Assessment initiated a plan to populate a publicly accessible dataset of control animal microarray data to serve as a resource for analysis of baseline fluctuations in gene expression due to biological or technical factors. Datasets from control animals within toxicogenomics study arms were solicited from HESI participants in the US and Europe. The dataset was limited to rat liver and kidney samples run on Affymetrix arrays in order to harmonize the appropriate data format and content for the dataset, and the feasibility of comparing signal data across multiple sites and conditions. Information was collected on common variables in toxicity studies (e.g., dosing regimen) and other known confounding factors that can affect sensitivity to chemicals in toxicity studies (strain, supplier, gender, diet, and age) [8]. In this paper, the collected control animal microarray data is analyzed for the contribution of different study factors to baseline variability in gene expression. Genes were identified which had the most and least inherent variability, were gender-selective, or altered by fasting.

## Results

### Dataset description

To populate a dataset of baseline gene expression, voluntary contributions of microarray data from the control arms of toxicogenomics studies of liver and kidney were requested from HESI member institutions. A survey form was developed and sent to contributors requesting metadata about the study including information on subject characteristics and husbandry, euthanasia methods, specimen preparation and preservation protocols, RNA preparation and labeling, and microarray hybridization (Table 1). On receipt of the data from contributors, terms were harmonized and entered into binned ranges where needed (e.g., age), and anonymized as to contributing institution.

**Table 1:** Study factors collected with control animal microarray data

**Category**	**Study Factor Query**
Study	Organism name
	Animal strain
	Sex
	Study number
	Institution

Subject characteristics	Individual animal identifier
	Animal supplier
	Animal age at sacrifice
	Total body weight at sacrifice
	Time of day at sacrifice (AM or PM)

Husbandry	Diet name and source
	Diet availability
	Number of animal in a cage
	Number of hours of light per day
	Was food withheld prior to sacrifice?
	Fasted > 12 hr?
	Blood collection during in life study?
	If yes, time of collection relative to sacrifice

Specimen	Organ Sampled
	Organ section sampled
	Organ preservation method
	Organ weight at sacrifice

Euthanasia	Sacrifice method
	Exsanguinated?
	Anesthetic used

Treatment	Vehicle
	Route of vehicle administration
	Frequency of dosing
	Duration of dosing
	Dose volume
	Time between dosing and sacrifice

Microarray experiment	RNA extraction protocol
	Quality parameters for RNA
	Sample preparation protocol
	Rounds of amplification
	Amount of RNA on array
	Array type used
	Scanner model

Signal data from a total of 536 microarrays were received from 16 institutions and 48 in-life studies. Each study contains a unique combination of treatments and handling conditions. The data was collected on 3 Affymetrix rat expression array types (RGU34A (n = 192), RAE230A (n = 213), and RAE230 2.0 (n = 131)) for two tissues, liver (n = 396) and kidney (n = 140). For further analysis, the data was partitioned into 6 tissue-array sets. The dataset also included 3 rat strains (Sprague-Dawley (n = 302), Wistar (n = 210), and F344/N (n = 24)) and both males (n = 436) and females (n = 100). A list of the 38 study factors requested with the data submissions is included in Table 1. Details on the distribution in the collected data of 22 of the 38 study factors are contained in Additional file 1. Complete information was not received for every study factor for all contributed microarray data files.

Fifty-three data files were excluded from further analysis because they were associated with factors known to impair data comparability (high photomultiplier tube setting on scanner, quality metric outlier) or that were identified to be duplicate submissions. The remaining 483 sets of array data were processed using Robust Multi-array Average (RMA) separately for each of the 3 array types in the dataset.

### Variability analysis

**Figure 1:**
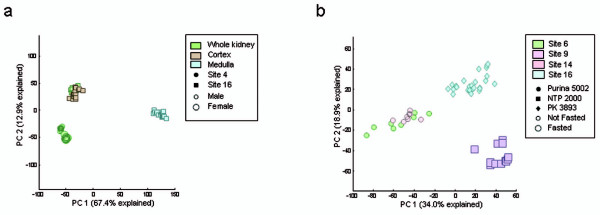
**Principal components analysis of control kidney and liver data**. Principal components were computed for normalized intensities from (A) kidney and (B) liver samples run on RAE230 2.0 arrays. The first two principal components are shown, which together explain about 80% and 53% of the total variation for kidney and liver, respectively. Each point in the plot represents an individual sample. (A) For kidney, organ section (indicated by color) appears to be the prominent explanatory factor. Lab is indicated by marker shape and gender is indicated by marker size. (B) Lab, diet, and fasting (indicated by color, shape, and size, respectively) are confounded in the liver dataset, and their respective levels are strongly associated with three distinct clusters. The sources and numbers of the 3 rat chows used in this dataset were Purina 5002, National Toxicology Program (NTP) 2000, and Provimi Kliba (PK) 3893.

To statistically assess prominent sources of variability in the data, we used several multivariate techniques, applied separately on each of the six tissue-array data subsets for 15 fully entered study factors and 2 study factors with partial entries (listed in Table 2). Within each subset, the genes were individually mean-centered, that is, the mean of the log2 intensity values for each gene was subtracted from each of its values, and principal components were computed. The first two components were plotted using different colors and markers to label the various factors in an ad hoc fashion. This approach enabled identification of several factors associated directly with clusters of principal component scores. For example, organ section was observed to be a prominent source of variability in the kidney RAE230 2.0 dataset (Figure 1A). In contrast, analysis of the liver RAE230 2.0 dataset was less straightforward because of confounding between sites, diet, strain, and fasting (Figure 1B). Principal component analyses of other tissue-array sets are available in Additional file 2. The principal component scores for these analyses are available at both the Chemical Effects in Biological Systems Biomedical Investigation Database (CEBS-BID) [9] and ArrayExpress at EBI [10].

**Table 2:** Summary of study factors with significant Hotelling-Lawley (H) and Variance Components (V) scores in each tissue-array set

	Liver RAE230A	Liver RAE230 2.0	Liver RGU34A	Kidney RAE230A	Kidney RAE230 2.0	Kidney RGU34A
Gender	H^a ^V^b^	-^c^	V	H V	V	^d^
Fasted	V	H V	V	-		
Strain	V	H		-		H V
Organ Section		H		-	HV	-
Fixation	V	-			-	H V
RNA Amount	V	H			-	H
Lab		H	H			H
Dose Duration		H V			H	
Vehicle	H	H				H
Sacrifice Method	V	H		-		
Diet		H V		-		
Study(Lab)			H			
Route			V		-	
Age						H
Dose Frequency	V			-	-	
Scanner		-	V	-	-	
Anesthetic				-		

^a^H: Large Hotelling-Lawley statistic (HL_i _> 35% of HL_full_)

^b^V: Large Variance Components statistic (V_i _> V_error_)

^c^"-" Not tested (single factor level within tissue-array set)

^d^Blank: Small effect for both H and V

To more formally assess contributions to variability, Hotelling-Lawley (HL) and Variance Components (VC) scores were computed for each of the 17 study factors in each six tissue-array set. HL statistics provide individual factor scores for the degree of variability explained, as compared to a maximal model that includes every distinct factor combination for that data subset. The significance of an individual effect according to HL does not necessarily indicate that a particular effect is a true source of variability, because it may be confounded with other effects, but it does indicate that at least one source is likely present. Variance Component (VC) scores are different from HL in that they sum to one and provide a simultaneous partitioning of total variability. VC scores are subject to a degree of indeterminability when there is complete confounding between two or more factors. In this case, the proportion of variability shared amongst confounded factors is assigned to factors in a way that is dependent on their order in the model and the specific REML algorithm employed. The total fraction of variability assigned to those factors is preserved, so where only one of the confounded factors is a true source of variability, the assigned VC score to it will tend to be biased downwards.

The results of the multivariate variability analyses when viewed in summary across the six tissue-array sets indicate that gender, fasting, strain, and organ section were the most reproducibly prominent biological factors associated with gene expression variance between control animals in this dataset (Table 2). While lab (or institution) was a clearly observable source of variance, it was always confounded by a unique combination of study factors used within each laboratory's in-life studies and possible contributions to variance from technical factors which cannot be discerned in this analysis. Since the collected data are observational (that is, not from a single designed experiment), extreme care must be taken in interpreting large scores because many factors are completely or partially confounded with others. In addition, the meaning of a particular factor can change from subset to subset, depending on its observed levels. Tabular displays of factor combinations alongside the HL and VC statistics are available in Additional file 3 to aid in discerning confounding relationships.

**Figure 2:**
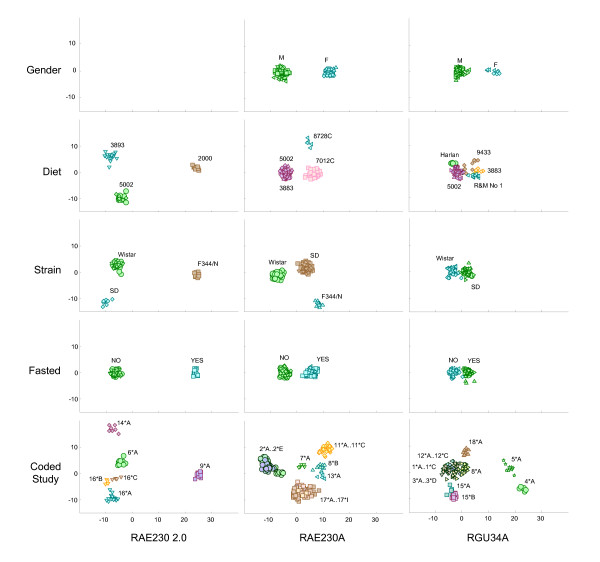
**Canonical variable plots of control liver data**. Each panel shows the first two canonical variables, which represent the maximum achievable separation for each factor (in rows) and array type (column), computed from the first ten principal components. Each point in the plot represents an individual sample. The marker color along with text indicates the factor level for the sample and the shape indicates the site where the data were generated. Coded study is indicated by site number followed by a letter indicating study within a site.

To further explore attributable variability, we performed a discriminant analysis on the following factors: gender, diet, strain, fasted, and coded study (nested within lab). The first ten principal components were used as predictors for this analysis. The resulting first two canonical scores for the three liver data sets are plotted and labeled in Figure 2. The figure reveals very clear clusters of points assignable to distinct levels of the different factors; however, it is important to realize that a confounded factor may in fact be the true cause for the clustering. Table 3 shows factors observed to be confounded with those analyzed in Figure 2. The confounding relationships were determined by fitting a partition tree model to each factor in Figure 2, using the other 16 factors as predictors. Additional files 4 and 5 contain a similar figure and table for kidney, and Additional file 3 details full interrelationships amongst factor levels.

**Table 3:** Confounding relationships for selected factors in liver

	RAE230A	RAE230 2.0	RGU34A
Gender	None	n/a – all males	None
Diet	OrganSection + Fixation	OrganSection + DoseDuration/Strain/SacMethod/Vehicle	None
Strain	OrganSection + Fixation	SacMethod + DoseDuration/RNAAmount/Age/Fasted/SacMethod/Diet/Vehicle/OrganSection	Diet + DoseDuration/Route/Age/OrganSection
Fasted	OrganSection or Diet + Strain	DoseDuration/Strain/SacMethod/Diet/Vehicle/OrganSection	Diet + RNAAmount + Scanner/Fixation/SacMethod/Vehicle/OrganSection

### Individual gene variance analysis

Variance in gene expression between individuals in control groups may be attributed to differences in environmental factors, genetic background, and measurement reliability. Gene variance that is consistently observed among control animals but cannot be readily attributed to any identified study factor can adversely affect the reliability and reproducibility of toxicogenomics data. To identify probes in this HESI project that had the highest and most consistent baseline variance not attributable to any known study factors, expressed probes in each tissue-array set with the highest baseline variance (top 2%) were intersected across arrays by probe ID. A subset of probes was identified for which there was replicate evidence of high variance (ranked by variance in the top 5% of expressed targets either across tissue-array sets or for multiple probe sets per array) or relevance to toxicogenomics (provisionally defined as inclusion on the Affymetrix GeneChip Rat ToxFX 1.0 array). Using these criteria, probe sets were identified for 373 targets that exhibited reproducible high baseline variance in liver (n = 103), kidney (n = 121), or both tissues (n = 149) (Additional file 6).

Gene pathways and functions most closely associated with high baseline variance were identified using annotation classification programs in the Database for Annotation, Visualization and Integrated Discovery [11]. Genes highly variant in control liver and kidney samples were enriched and uniquely present in the KEGG pathways (EASE score < 0.05) for "antigen processing and presentation" (major histocompatibility complex genes), "androgen and estrogen metabolism" (*Hsd11b1, Hsd11b2, Hsd17b3, Srd5a1, Ugt2b4*), "maturity onset diabetes of the young" (*Foxa2, Foxa3, Gck, Onecut1*), "metabolism of xenobiotics by cytochrome P450" (*Adh1, Adh4, Cyp2c40, Cyp3a13, Cyp3a18, Cyp4a14, Ephx2*), "glycine, serine, and threonine metabolism" (*Agxt2, Alas2, Chka, Dao1, Phadh, Sds*), and "biosynthesis of steroids" (*Hmgcr, Idi1, Ngo1, Sqle*). Altogether, these six pathways include 43 of the high variance genes (see Additional file 6).

Functional annotation clustering analyses using gene ontology (GO) terms was performed to identify enriched molecular functions and biological processes among the remaining high variance genes. High variance genes (53 total) were enriched in four molecular function clusters associated with (1) monooxygenase or other heme binding activity (*Cyp2b2, Cyp2d26, Cyp2d9, Cyp2d10, Cyp4b1, Cyp7a1, Cyp8b1, Cyp17a1, Hbb*), (2) cytokine, growth factor, or other receptor binding activity, (3) carbohydrate binding (*Apcs, Lpl, TSP-2*), and (4) transporter activity. High variance genes (120 total) were also enriched in three clusters of biological processes associated with the molecular functions of (1) lipid metabolism, (2) defense response (*C3, Ctgf, Hamp, Itga1, Lbp, Spp1, Vwf*), and (3) transport (*Acadsb, Akr1c6, Apoe, Cd36, Cyb5, Cyp17a1, Cyp7a1, Cyp8b1, Egf, Egfr, Fasn, G6pc, Hspb1, Igf1, Pck1, Pik3r1, Ppp1r3c, Ptgds, Sc4mol, Slc7a7, Srebf1, Tgfb2*). Enriched GO terms and other annotations are listed in Additional file 6 for the high variance genes.

Expressed genes with low inherent variability are of interest in toxicogenomics data analyses for data normalization or, if significantly changed in response to exposure or damage, as potentially reliable and specific biomarkers. Probe targets with multiple instances of low overall variance in liver, kidney, or in both tissues were identified across tissue-array sets. A combined non-redundant list of 163 genes with the lowest variance in control animals was compiled for functional annotation analysis (Additional file 7). Interestingly, this list contains 8 genes expressed in both liver and kidney (*Cyp2d26*, *Tf*, *C3*, *F2*, *ApoE*, *Spp2*, *Rbp4*, Rn.107069) that were highly variant in control kidney, but among the least variant in control liver samples. The low variance gene list was enriched (enrichment scores > 1.5) for genes annotated with the GO cellular component terms of mitochondrion or other organelle membrane and for biological process clusters primarily associated with transport or defense response. Many of these genes identified with low overall variance are involved in housekeeping functions often associated with controls for gene expression studies. Examples include *Actb, Gapd, Aldoa, Pgam1*, and *Rps29 *which are commonly used as control genes in mammalian expression studies [12].

### Gender-selective genes in liver and kidney

**Figure 3:**
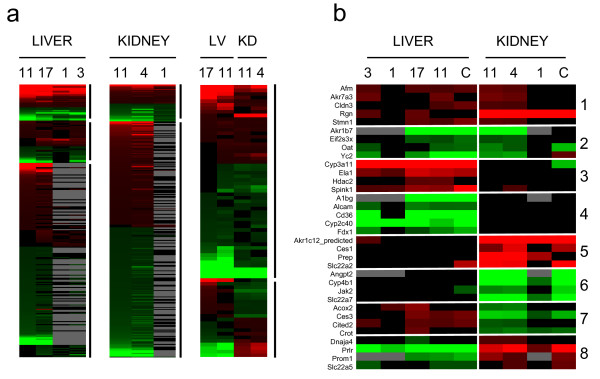
**Heat map visualizations of gender-selective genes in liver and kidney**. The color scale indicates the ratio of expression in males relative to females in the visualizations created using default settings in TreeView [32]. Red indicates higher average expression in males, green indicates higher expression in females, black indicates no significant difference between males and females, and gray indicates that no data was available due to the smaller genome coverage by the RGU34A arrays used by institutions 1 and 3 (numbers above columns indicate the institution that contributed the data). All other institutions used RAE230A or RAE230 2.0 arrays. (A) The left panel shows liver selective genes with a ratio range of 200 to -190, the middle panel shows kidney selective genes with a ratio range of 113 to -89, and the right panel shows gender-predominant genes that have the same or opposite direction of expression in liver and kidney over a ratio range of 81 to -190. Only those genes that were altered in at least two-thirds of sites for kidney, at least half of the sites for liver, or three-quarters or more of the sites in cross-tissue comparisons are shown. (B) Verification of identified classes of gender-selective genes. Genes that exhibited significantly altered expression between males and females in liver or kidney were divided into 8 different groups based on expression behavior as indicated on the right side of the panel. Groups 1, 2, 7 and 8 exhibited gender-selective expression in both liver and kidney. Groups 3 and 4 exhibited gender-selective expression predominantly in liver and groups 5 and 6 exhibited gender-selective expression predominantly in kidney. The expression of four to five genes from each of the groups was examined by RT-PCR (column C) in liver and kidney from control male and female F344 rats from an independent study. The relative ratios span a range from 300 to -267.

To determine the dataset's utility for suggesting genes associated with different experimental factors, we identified genes that were differentially expressed between males and females (gender-selective genes) using EPIG, a profile-based analysis method for Extracting microarray gene expression Patterns and Identifying co-expressed Genes [13]. This analysis involved transcript profiles from 14 studies carried out at 5 institutions and included datasets from 76 male and 58 female rat livers and 30 male and 28 female rat kidneys (see Additional file 8). A total of 854 or 863 genes exhibited significant differences in expression levels between the genders in one or more institutions in liver or kidney, respectively (see Additional file 9). About 30% of these genes exhibited significant differences across more than one institution (Figure 3A, left and middle panels). Overall, 265 gender-selective genes in liver and 305 genes in kidney showed excellent concordance between institutions in terms of gender-selective expression as well as magnitude of the difference between genders. While 76 genes consistently exhibited similar gender-selective behavior in both liver and kidney (Figure 3A, right panel), 22 genes showed reverse patterns of gender-selective expression between liver and kidney. For example, *Ces3 *was predominantly expressed in males in liver and in females in kidney, while *Prlr *exhibited the reverse behavior.

Genes that exhibited gender-selective expression in liver or kidney were divided into 8 different groups based on expression behavior (Figure 3B). Genes were identified that were male-predominant (group 1) or female-predominant (group 2) in both liver and kidney, male-predominant in liver only (group 3) or kidney only (group 5), female-predominant in liver only (group 4) or kidney only (group 6), male-predominant in liver but female-predominant in kidney (group 7) or vice versa (group 8). The relative expression levels of 4 to 5 genes in each group were examined by RT-PCR in liver and kidney from control male and female rats from an independent study using the F344/N strain (Figure 3B). Although the EPIG analysis was performed using data from Wistar and Sprague-Dawley rats and the RT-PCR results were generated in F344/N rats, there was generally good agreement between the microarray and RT-PCR results, confirming the robustness of the methods used to identify gender-selective genes (Additional file 10).

The gender-selective genes fell into a number of functional categories including those involved in "xenobiotic metabolism" and "response to xenobiotic stimulus" (see Additional file 11). Many of the genes in this category are classic sexually dimorphic genes that have been previously characterized, including members of the CYP2A, CYP2C, and CYP3A families [14]. Liver and kidney gender-selective genes also included those involved in "cellular lipid metabolism", "lipid metabolism" and "fatty acid metabolism," a number of which were identified in two microarray studies of gene expression in the livers of male and female rats [15-17]. Gender-selective genes in kidney also included those involved in metabolism and transport of other metabolites including "aromatic compound metabolism," "carboxylic acid metabolism," and "organic acid metabolism." The 12 named genes that exhibited opposite regulation in liver and kidney included those involved in estrogen metabolism (*Hsd17b2*), xenobiotic metabolism (*Sult1c1*), lipid metabolism (*Acox2*, *Crot*, *Pld1*, *Dgat2*), transcriptional regulation (*Cited2*), and signaling (*Prlr*, *Prom1*).

The gender-selective genes identified in liver were evaluated for common regulatory elements in their promoters. Three classes of transcription factor binding sites were identified. Many of the genes contained STAT1, STAT3, STAT5, and STAT6 sites. STAT1, STAT3, and STAT5 are activated by growth hormone (GH) and STAT5b plays a major role in determining gender-selective gene expression through GH-janus kinase pathways [14]. Other sites included those recognized by the LXR-RXR, FXR-RXR, or VDR-RXR nuclear receptor heterodimers that respond to oxysterols, bile acids, and vitamin D, respectively. The gender-selective genes also had a significant number of sites recognized by CCAAT displacement protein family members including Cut and Cux which are homeodomain-containing proteins that repress the transcriptional activities of HNF-1α, C/EBPα and Rb/p107 and have not been previously associated with gender-selective gene expression.

### Genes changed by fasting in liver and kidney

**Figure 4:**
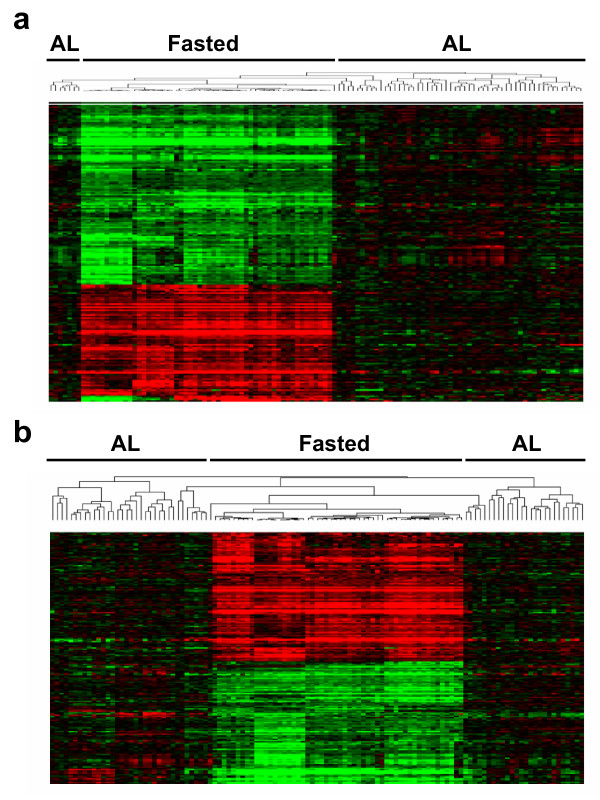
**Two dimensional hierarchical clustering of genes that exhibited altered expression in liver samples from *ad libitum *(AL) fed or fasted rats**. Genes that were significantly changed by overnight fasting were identified using either (A) EPIG (n = 190 genes) or (B) t-test (n = 311 genes) analysis of data from 115 cel files and 5 sites. Gene expression levels were normalized to the average AL fed value and color-coded on a fold change scale of 6.4 to -9.5 (red to green), using default contrast settings in TreeView [32].

The control animal dataset was also used to identify genes that were regulated by fasting. Although not specified, fasting was likely to have been ~12 to 18 hr in duration, which is typical for studies examining the effects of fasting on organ function. The transcript profiles from a total of 11 studies carried out at 5 institutions were used to identify genes altered by fasting (Additional file 8). This dataset included 115 .cel files for liver samples from 85 males and 30 females from three strains of rats, with 61 animals fed *ad libitum *(AL) and 54 animals fasted. Genes associated with fasting were identified by EPIG and t-test as described in the Methods. There were a total of 190 or 311 genes that were significantly different using EPIG or t-test, respectively, with 95% concordance between the two methods (Additional file 12). Two-dimensional clustering was used to determine the similarity of the gene expression profiles from the different animals (Figure 4). Although most of the animals clustered into two distinct clades for *ad libitum *fed and fasted, there were a number of AL animals that routinely clustered with the fasted animals. These animals included those from institution 11 (studies A and B) and institution 2 (studies D and E). An examination of the expression profiles from these animals indicated that a number of the fasting responsive genes were altered, although, in most cases, not to the extent observed in the fasted animals. Thus, it is possible that these animals were subjected to fasting conditions for short duration either intentionally or because of housing conditions.

Functional categorization showed that fasting altered the expression of the same categories of genes that had previously been identified in mouse liver [18] and that are dominated by genes involved in lipid utilization (Additional file 13). It should be noted that no global analysis of fasting-responsive genes in rat liver has been published. However, the impact of fasting on the interpretation of toxicogenomics studies has been discussed [19,20].

Genes differentially regulated by gender or fasting state also tended to have high baseline variability. Approximately 34% of the gender and fasting genes are included in the 500 RAE230A probe sets that have the highest baseline variability (3% of the total array content) and all gender or fasting genes are included among the top ~31% most variant probe sets (Additional file 14). This indicates that genes that respond to fasting, presumably through nutritional cues, and those gender-predominant genes that are under control of growth hormone substantially contribute to variability in a liver gene expression profile.

## Discussion

Through the consortial efforts of participants in the HESI Committee on Genomics, a unique resource has been assembled that connects gene expression levels in the liver and kidney of rats from the control arms of numerous, diverse toxicogenomics studies to specific study meta-data. This dataset and the complete results of variability analyses described in this manuscript are available for further data mining in the CEBS-BID and EBI ArrayExpress public databases. One outcome from this project is the identification of key descriptors that should be included in the minimal information about a toxicogenomics study needed for interpretation of results by an independent source. Among the key descriptors are those that are prominent sources of variability in baseline gene expression.

Multivariate variability analysis of the baseline expression dataset revealed gender, fasting, and organ section as some of the most prominent factors associated with overall transcriptional changes, although most of the seventeen factors included in the multivariate analyses appeared to be a significant source of variability in at least one of the six tissue-array datasets. Fasting tended to be a stronger source of variability in liver than kidney, while organ section was a more prominent source of variation in kidney than liver. In agreement with a recent study that examined gene expression profiles in kidney slices [21], we observed that the cortex was more similar to whole kidney than the medulla.

The observational nature of this study severely hinders causal inference, but the results can provide at least some positive associations of particular factors of interest. For example, unambiguous gender and organ section differences were discernable. On the other hand, while it is hypothesized that certain variables within a study factor field may contribute disproportionally to the observed variance (e.g., the use of oil as a vehicle or a diet lower in protein or phytoestrogens), many of these could not be confirmed with the current dataset because of confounding with other factors. Hypotheses generated from these data could be tested by experiments specifically designed to test the variability due to specific factors of interest.

Many of the genes identified as having high baseline variability have relevance to toxicology. This list includes many genes involved in the absorption, distribution, metabolism, and excretion of drugs (e.g., members of the ABC and SLC transporter superfamilies, phase I and II enzymes) and is also enriched for genes involved in defense response. Similar results were observed in a recent study of variability in the human liver transcriptome [22]. Among 75 diverse individuals, the most variable hepatic genes were involved in drug and intermediary metabolism, inflammation, and cell cycle control. One contributing factor to high baseline variability may be increased sensitivity in transcriptional response to environmental cues. Therefore, it follows that some of most variable genes in control animals could also be significantly changed by exposure to drugs or chemicals. As an example, we compared the list of gene transcripts with high baseline variance (in Additional file 6) to significant gene changes observed across laboratories in collaborative research studies conducted by the HESI Committee on Genomics [23]. About 20 percent of the genes associated with renal injury by cisplatin across multiple platforms (*Col1a1, Egf, G6pc, Igfbp3, Lcn2, Rpb4, Spp1, Ugt2b*) [24] or significantly regulated in liver by the hepatotoxin clofibrate (*Cyp17a1, Hspa1a, Cpt1a, Egr1*) [25] were also identified to have high baseline variance in this study. However, genes with high baseline variability may be identified as statistically different between treatment groups of small size without actually being changed by treatment.

To illustrate the utility of the multi-site control animal dataset for identifying gene changes associated with specific study factors, we focused on two factors, gender and fasting, that had the greatest contribution to variance in the largest number of tissue-array sets (see Table 2). Although strain and organ section also ranked high and are important variables in toxicogenomics studies, an analysis of gene changes associated with these factors was not included in this manuscript because of certain constraints with the available data sets. For example, fasting was confounded by strain in the Liver-RAE230 2.0 set and by strain and gender in the Liver-RAE230A set. We did not consider liver section to be a well standardized term based on the responses received. Although kidney section was clearly associated with significant differences in gene expression levels, only one site contributed data for different kidney sections. Investigators are encouraged to use the control animal dataset for exploratory analysis of genes changes associated with strain or tissue section.

The dataset showed utility for identifying genes that exhibit differential expression in males and females as well as genes that are altered by fasting. The large number of rats in the analysis (192) provided a unique statistical power to identify novel genes that exhibit gender-selective gene expression and can be used for further elucidation of regulatory control elements, some of which may depend on tissue context. Identification of differences in gene expression between genders in tissues that are important in the response to and metabolism of pharmaceuticals or environmentally relevant chemicals will be useful for understanding the functional basis of gender differences in the pharmacology and toxicology of these compounds.

In planning microarray expression experiments, this paper highlights a number of concerns that an investigator should consider in defining a new experiment or in comparisons with reported results. Gender, strain, diet, and tissue section within an organ can all play a role in defining baseline gene expression. In the case where known experimental factors are controlled, there are genes that show inherent variability. Future experiments could be designed to uncover the factors that can account for this variability. Alternatively, variability in expression may be due to other biological factors that were not identified here. In designing an experiment with few biological replicates, one of these highly variable genes may randomly partition into an experimental group leading to an attribution of the change in gene expression to an experimental factor. The online and supplementary materials can be used to identify genes which are significantly altered by external factors as well as genes which have high inherent variability.

## Methods

### Data access

The raw data (.cel files), RMA normalized data, MAS 5.0 processed data, and report files are available at the EBI ArrayExpress database [10] under accession number E-TOXM-39. In CEBS-BID [9], the data are provided in a single investigation ("HESI Baseline", Accession 008-00003-0001-000-2), containing 18 studies, each corresponding to a contributing laboratory. Within each laboratory, individual in-life studies are represented by groups.

### Array outlier analysis and data normalization

Several steps were taken to ensure data consistency across datasets contributed by different institutions. First, arrays (n = 34) scanned using high PMT settings were ignored for better inter-array comparability. Second, duplicated .cel files (n = 2) with identical data contents but different experimental annotation were identified by pair-wise comparison and removed. Third, for the remaining 500 scans, Affymetrix .rpt files, generated from processing .cel files using MAS 5.0 software [26] with total chip intensities scaled to an average intensity of 250 and with manufacturer-defined default parameters, were analyzed to further identify quality outliers. The quality metrics analyzed from .rpt files include percentage of present calls, scaling factor, average and maximum background, as well as 3' to 5' signal ratios for the Affymetrix control transcripts. Scans (n = 17) exceeding arbitrary cutoffs for scaling factor (>10), average background (> 120), or maximum background (> 130) were excluded from further analysis. Because measures of different quality metrics correlate with each other, the excluded outliers also had significantly lower percentage present calls and higher 3' to 5' signal ratios for glyceraldehyde-3-phosphate dehydrogenase and β-actin, as compared to the retained arrays.

The 483 arrays remaining after outlier exclusion were processed using RMA. For each of the three separate RMA runs corresponding to the three different array types, the individual perfect match (PM) probe values were background-corrected and quantile normalized before computing the expression values, as described by Irizarry *et al*. [27]. Besides using RMA as our default normalization method, we also assessed the impact of normalization on data analysis by comparing RMA results with results derived from MAS 5.0 and dChip [28]. Only marginal difference was observed in outcomes among the three different normalization methods, including correlation coefficients of the expression values and agreement of lists of significant genes identified using statistical tests.

### Variability analysis

Hotelling-Lawley (HL) statistics [29] were computed individually for each of the 15 factors for which there is complete (no missing) data and for two factors with partially filled values (Organ Section, which has 5 missing values in the RGU34A Liver subset and Scanner which contains 104 (out of a total of 483) values that were coded as 0 (unknown)). Studies that were submitted from different sites within the same parent institution were coded as separate laboratories. Sites 12 and 15 represent different research centers within one company, as do sites 3 and 5. HL was computed using the first ten principal components as a multivariate response, and then standardized to be between 0 and 1 by dividing by HL statistic for the maximal model consisting of all distinct factor combinations. A threshold for significance of the HL statistics was set empirically at 0.35.

To provide a competitive partitioning of sources of variability, a variance components (VC) model was fitted separately to each of the first ten principal components. Considering a particular principal component score vector to be a univariate response, the 17 aforementioned factors were modeled together as Gaussian random effects on that response and a variance component was estimated for each, using restricted maximum likelihood [30]. The model is invariant to ordering of the effects, except when there is complete confounding. Because of the possibility of confounding for certain data subsets, the effects for Lab and Study(Lab) were always specified last in the model in order to give the other, known, confounded sources of variability priority in explaining variability. The ten REML variance component estimates for each factor were averaged, using the corresponding eigenvalues as weights. A weighted-average REML estimate of residual (unexplained) variability was also computed. The final set of weighted-average estimates were divided by their sum to yield a proportion-of-total-variability estimate for each factor and the residual variance. The residual error was the cutoff for significance for a VC statistic.

The first ten principal components for each of the six data subsets were also used as predictors in a classical linear discriminant analysis (LDA) [29]. LDA was conducted separately on the following factors: gender, diet, strain, and fasting, as well as study(lab) as a reference. Canonical scores from this analysis are based on the eigen-decomposition of a function of the between and within cross-products matrices of the corresponding multivariate analysis of variance. The canonical scores represent directions of maximal separability for the factor levels.

The same four factors were also predicted using other factors in order to discern confounding patterns. A partition tree (recursive partitioning) algorithm was used here. In a few cases (e.g. gender), no obvious confounding was apparent, which provides support in these cases that this factor was in fact the true source of variability. In other cases, many factors were completely confounded, making it difficult to pinpoint true sources.

The preceding variability analyses were performed using MatLab and JMP Genomics software.

### Real-time RT-PCR analysis

The relative levels of expression of selected genes were quantified using real-time RT-PCR analysis. Tissues were derived from an independent study carried out at the US EPA using 8–9 week old male and female F344/N rats. Only control rats were used to validate microarray results. Liver and kidney RNA was isolated using a modified guanidium isothiocyanate method (TRIzol^®^, Invitrogen, Carlsbad, CA) and was further purified using silica membrane spin columns (RNeasy^®^, Qiagen, Valencia, CA). RNA quantification was determined using a Nanodrop spectrophotometer and RNA integrity was assessed by the RNA 6000 LabChip^® ^Kit using a 2100 Bioanalyzer (Agilent Technologies, Palo Alto, CA). Total RNA was reverse transcribed with MuLV reverse transcriptase and oligo-dT primers. The forward and reverse primers for selected genes (see Additional file 15) were designed using Primer Express software, v2.0 (Applied Biosystems, Foster City, CA). The Power SYBR^® ^Green PCR Master Mix (Applied Biosystems, Foster City, CA) was used for real-time PCR analysis. Reactions were run in duplicates and repeated twice. The Ct values of the genes were first normalized to β-actin and 18s rRNA levels in the same sample, and then the relative differences between control and treatment groups were calculated and expressed as relative increases, setting the control as 100%. Means and S.E. (n = 3) for RT-PCR data were calculated by Student's t test. The level of significance was set at P ≤ 0.05.

### EPIG analysis

EPIG extracts a set of patterns representing co-expressed genes without pre-defined seeding of the patterns [13]. The method uses gene expression profile Pearson correlation measures, magnitude of change, and signal-to-noise evaluations to categorize genes into patterns that represent co-expressed genes. A 2-dimensional matrix of microarray gene expression data (log_2 _pixel intensity ratio values) with each row representing a gene expression profile and each column representing an array is used for analysis. A gene expression profile can be made between and within groups. The arrays in within-groups have a factor in common, e.g. biological replicates. The arrays in between-groups possess different factors, e.g., fasting or gender. We denote each data of log_2 _ratio as g_ij _in a gene expression profile, where i refers to a between-group index from 1 to m, j is the within-group index from 1 to n_i_, m is the number of between-groups and n_i _is the number of arrays in i^th ^between-group.

Briefly, using all pair-wise correlations, any candidate profile whose local cluster size is less than a predefined size M_t _or correlation is higher (> R_t_) but has a lower relative local cluster size M is removed from pattern construction consideration. EPIG then creates representative profiles for the corresponding local clusters. Lastly, candidate profiles with a signal-to-noise ratio < 3 (the number of time the signal is above the noise) or its magnitude of expression S < 0.5 is removed from consideration in the pattern building process. Subsequently, EPIG categorizes each of these genes to one of the patterns, with which it has the highest correlation value of the gene profile. A gene not assigned to any extracted patterns is considered an "orphan" if its highest Pearson correlation r-value is lower that a given threshold R_c_. Typically R_c _is set to a value which corresponds to a correlation p-value of 10^-4 ^to assure the significance of the co-expression. For this analysis, M_t _was set to 6 and R_t _set to 0.8.

Genes were selected with the following cut-offs: Log_2 _ratio ≥ 0.5 (or ≤ -0.5) and p-value ≤ 0.001. Biological analysis was performed using the Gene Ontology function of GeneGo with the help of KEGG [31]. In the figures and additional files for genes selective for gender or fasting, fold change is reported as the negative reciprocal value if less than one. Analysis of regulatory elements within the promoters of gender-selective genes was performed using Genomatix software (Genomatix Software GmbH, Munich, GER).

### Probe set variability analysis

A baseline variance was calculated for each probe set within each tissue-array set based on the maximal model, in which each unique combination of factors was encoded as a distinct group. These values are available through CEBS-BID and EBI. Variance within each group is interpreted as baseline variability and is not due to any of the study factors for which information was collected.

In order to define whether a probe set is detectably expressed, the signal strength of perfect match (PM) probes was compared to the signal strength of mismatch (MM) probes in a two way test for equal intensity using the log of the baseline adjusted intensities. Probe sets with PM ≠ MM signals (P < 0.05) in ≥ 80% of samples per tissue-array set were defined as expressed in a given tissue in this study. The fraction of probe sets with expressed signals ranged from 30% (in the liver-RGU34A set) to 50% (for the kidney-RAE230A set) of the total number of probe sets on each array type. Probe sets were matched between the RGU34 and RAE230 series using "Best Match" sequence mapping files available from the manufacturer or by UniGene identifier (July 2006 update). To identify genes with multiple instances of low overall variance, the 200 least variant probe sets in each tissue-array set were intersected by probe identifier.

Overrepresentation of biological themes among most and least variant genes was performed in DAVID using functional annotation clustering analysis [11]. The 8630 probe sets expressed in liver or kidney on the RAE230A array were used as the population background for enrichment analysis. Enrichment analysis was performed for annotation clustering by KEGG pathway, Gene Ontology (GO) Molecular Function Levels 2 and 3, GO Cellular Component Levels 3 and 4, or GO Biological Process Levels 3 and 4. The threshold for labeling an annotation cluster as enriched was an enrichment score > 1.5.

## Authors' contributions

MJB contributed to the data analysis, writing, and editing of the manuscript. RDW contributed to the variance components analysis. MBB contributed to the writing and editing of the manuscript. PRB drafted the methods section of the manuscript relating to the EPIG analysis. JWC performed the EPIG analysis of the data. MC assisted in the initial stages of the project defining goals and scope, and in the QC and normalization sections. JCC analyzed data on the gender and fasting genes, interpreted results, and wrote a section of the manuscript. JF developed the database resource to house and QC the data, and oversaw data management. SH identified transcription factors that potentially regulate the gender and fasting genes, interpreted results, and wrote a section of the manuscript. JSL analyzed processed data on the gender and fasting genes, interpreted results, and wrote a section of the manuscript. FL participated in the QC analysis, performed data normalization, and co-wrote a section of the manuscript with JQ. JL performed the RT-PCR experiments, interpreted results, and wrote a section of the manuscript. H–RQ contributed to the data analysis, writing, and editing of the manuscript. JQ contributed to the data analysis and development of analytical approaches. SP provided overall program management for the program design, data collection and analysis, and helped edit the manuscript. KLT contributed to the probe set variability analysis and helped draft and edit the manuscript.

## Supplementary Material

Additional file 1 **Distribution of responses for 26 study factors that annotate the 483 control animal .cel files analyzed in this manuscript**. This file contains a tabulation of the responses reported on the survey forms that were received for each submitted Affymetrix .cel file for a control animal sample from toxicogenomics studies conducted at each institution. The fractional count per response is based on the total number of responses received, which were not complete for every study factor. Some responses were harmonized or binned in order to facilitate analyses of study factor variability. Click here for file

Additional file 2 **Principal component analyses of tissue-array sets**. Principal components were computed for normalized intensities from samples of given tissue-array combinations. Plots of the first two principal components are shown with the axis indicating the amount of variance in each component as a percentage of the total. Click here for file

Additional file 3 **Tabular display of study factor combinations alongside the HL and VC statistics for each tissue-array set of control data**. Hotelling-Lawley (HL) and Variance Components (VC) scores were computed for each of 17 study factors in each six tissue-array set. Scores in bold were above the threshold for significance. A threshold for significance of the HL statistics was set empirically at 0.35. The residual error was the cutoff for significance for a VC statistic. Click here for file

Additional file 4 **Canonical variable plots of control kidney data**. Each panel shows the first two canonical variables, which represent the maximum achievable separation for each factor (in rows) and array type (column). Each point in the plot represents an individual sample. The marker color along with text indicates the factor level for the sample and the shape indicates the site where the data were generated. Click here for file

Additional file 5 **Confounding relationships for selected factors in kidney**. This table shows factors observed to be confounded with those analyzed in Figure 2. The confounding relationships were determined by fitting a partition tree model to each factor (gender, diet, strain, or fasted), using the other 16 factors as predictors. Click here for file

Additional file 6 **List of Affymetrix probe sets associated with high variance in control rat liver and/or kidney**. This list contains 373 genes with replicate evidence of high baseline variance among the six tissue-array sets. Baseline variance was calculated for each probe set (within each tissue-array set) based on the maximal model, in which each unique combination of factors was encoded as a distinct group. Using these variance measurements, probe sets that were ranked in the top 2% in baseline variance among expressed targets were intersected across tissue-array sets by probe identifier. The intersected list was then filtered for probe sets that were among the top 5% most variant on at least a second tissue-array set, or that were relevant to toxicogenomics (defined here as inclusion on the Affymetrix GeneChip Rat ToxFX 1.0 array). Column A: Probe set identifier on Affymetrix RAE230 and ToxFX arrays, Column B: Probe set identifier on Affymetrix RGU34 arrays, intersected by Affymetrix Best Match sequence mapping file or UniGene ID (grey background), Columns C-E: UniGene identifier, Gene Name, and Gene Symbol, Column F: Tissue where high variance observed; Liver (L), kidney (K), or both liver and kidney (B), Column G: Inclusion on the Affymetrix ToxFX 1.0 array: Yes (Y) or No (N), Column H: Functional annotation associated with the probe set, identified using the following sources of curation in DAVID [11], KEGG: Kyoto Encyclopedia of Genes and Genomes pathway; BP: Gene Ontology Biological Process; MF: Gene Ontology Molecular Function; CC: Gene Ontology Cellular Compartment; COG: Clusters of Orthologous Groups of Proteins; SP: Swiss Prot knowledgebase ; IP, Interpro database. Click here for file

Additional file 7 **List of Affymetrix probe sets associated with low variance in control rat liver and/or kidney**. This list contains 163 genes with replicate evidence of low baseline variance among the six tissue-array sets. Baseline variance was calculated for each probe set (within each tissue-array set) based on the maximal model, in which each unique combination of factors was encoded as a distinct group. Using these variance measurements, 200 probe sets with the lowest baseline variance among expressed targets were intersected across tissue-array sets by probe identifier. The intersected list was then filtered for probe sets that were among the 200 least variant on at least a second tissue-array set. Column A: Probe set identifier on Affymetrix RAE230 and ToxFX arrays, Column B: Probe set identifier on Affymetrix RGU34 arrays, intersected by Affymetrix Best Match sequence mapping file or UniGene ID (grey background), Columns C-E: UniGene identifier, Gene Name, and Gene Symbol, Column F: Tissue where high variance observed; Liver (L), kidney (K), or both liver and kidney (B), Column G: Functional annotation associated with the probe set, identified using DAVID [11]. Click here for file

Additional file 8 **Sources of data used to identify gender-specific and fasting-related genes**. The institution number, study code, and key descriptors for the data sets used in the EPIG analyses of genes associated with gender-selective expression or with expression changed by fasting state are listed. Study codes were assigned to distinguish independent studies submitted from the same institution. Click here for file

Additional file 9 **List of probe sets identified as gender-selective using EPIG**. This file contains lists of gender-selective probe sets on Affymetrix RAE230 series and RGU34A arrays and the average fold-change difference within each tissue array set used in the analysis (male to female expression ratio). Fold changes less than one are reported as negative reciprocal fold changes. A list of probe sets with correlative and anti-correlative gender-selective expression between liver and kidney is also provided. Click here for file

Additional file 10 **Results of real-time RT-PCR analysis of gender-difference genes in livers and kidneys of F344/N rats**. Gender-selective expression of a subset of genes identified by EPIG analysis of the control animal dataset was verified in an independent study using RT-PCR. Each RT-PCR experiment was run twice with similar results. The expression levels in each animal were evaluated in duplicate, with 3 animals per group. Click here for file

Additional file 11 **Gene ontology analysis of gender-specific genes in the rat liver and kidney**. Results of analysis performed using the Gene Ontology function of GeneGo with the help of KEGG [31]. The max(-log(p-value)) is the result of a hypergeometric test using GeneGo. The higher the score, the greater the significance of the network. Network objects refers to the number of objects identified divided by the total number of objects in the network in the GeneGo database. Click here for file

Additional file 12 **List of probe sets identified as altered by fasting using EPIG or a t-test**. This file contains lists of Affymetrix RAE230 series probe sets that were significantly different in expression between fasted and *ad libitum *fed rats in the control animal dataset. For each probe set, the gene symbol, name, test statistic, and average fold change difference between fasted vs. non-fasted rats are provided. Fold changes less than one are reported as negative reciprocal fold changes. Click here for file

Additional file 13 **Gene ontology analysis of fasting-associated genes in the rat liver identified using a t-test or EPIG**. Results of analysis performed using the Gene Ontology function of GeneGo with the help of KEGG [31] is the result of a hypergeometric test using GeneGo. The higher the score, the greater the significance of the network. Network objects refers to the number of objects identified divided by the total number of objects in the network in the GeneGo database. Click here for file

Additional file 14 **Comparison of gender-specific and fasting genes to genes with the highest baseline variability**. This table contains a comparison of probe sets in common between a list of RAE230A probe sets ranked in order of highest to lowest baseline variability and lists of probe sets regulated by gender or fasting. Click here for file

Additional file 15 **Primer sequences for real-time RT-PCR analysis of gender-selective genes**. This file lists the gene symbol, GenBank number, and forward and reverse primer sequences for the 34 test genes and 2 control genes used to verify gender-selective expression. Click here for file
