# Supplementary material for: Sources of variation in baseline gene expression levels from toxicogenomics study control animals across multiple laboratories
Source: BMC Genomics. 2008 Jun 12;9:285. doi: 10.1186/1471-2164-9-285 (PMC2453529; doi:10.1186/1471-2164-9-285)
Supplement: Additional file 1 — Distribution of responses for 26 study factors that annotate the 483 control animal .cel files analyzed in this manuscript. This file contains a tabulation of the responses reported on the survey forms that were received for each submitted Affymetrix .cel file for a control animal sample from toxicogenomics studies conducted at each institution. The fractional count per response is based on the total number of responses received, which were not complete for every study factor. Some responses were harmonized or binned in order to facilitate analyses of study factor variability. [file 1471-2164-9-285-S1.pdf]

Study Factor	Level	Count	Fraction
**Array Type**	RAE230 2.0	117	0.24
	RAE230A	212	0.44
	RGU34A	154	0.32
	Total	483	1
**Sex**	F	98	0.20
	M	385	0.80
	Total	483	1
**Rat strain**	F344/N	23	0.05
	Sprague-Dawley	261	0.54
	Wistar	199	0.41
	Total	483	1
**Animal supplier**	Charles River	367	0.80
	Harlan	66	0.14
	Harlan Netherlands	11	0.02
	M&B A/S, Denmark	5	0.01
	Taconic Farms	12	0.03
	Total	461	1
**# of rats per cage**	1	254	0.57
	2	22	0.05
	3	3	0.01
	4	82	0.18
	5	84	0.19
	Total	445	1
**Binned Age**	< 6 wks	9	0.02
	6-7 wks	27	0.06
	7-8 wks	38	0.08
	8-11 wks	90	0.19
	10-12 wks	197	0.41
	> 12 wks	122	0.25
	Total	483	1
**Diet**	NTP 2000	12	0.02
	3883 (Provimi Kliba)	25	0.05
	3893 (Provimi Kliba)	68	0.14
	5002 (Purina)	270	0.56
	7012C (Harlan-Teklad)	68	0.14
	8728C (Harlan-Teklad)	5	0.01
	9433 (Nafag)	18	0.04
	R&M No 1	12	0.02
	Harlan	5	0.01
	Total	483	1
**Diet Availability**	ad lib	366	0.96
	restricted	16	0.04
	Total	382	1
**Vehicle**	acetone	10	0.02
	oil	45	0.09

	PEG400	17	0.04
	Polysaccharide*	285	0.59
	PS80	16	0.03
	saline	37	0.08
	untreated	25	0.05
	water	48	0.10
	Total	483	1

**Includes acacia, CMC, HPMC, MC, natrosol, and ora_plus*

**Vehicle Route**	feed	10	0.02
	gavage	337	0.70
	IM	6	0.01
	IP	21	0.04
	IV	10	0.02
	None	23	0.05
	PO	73	0.15
	SC	3	0.01
	Total	483	1
**Frequency of Dosing**	untreated	35	0.07
	once daily	423	0.88
	twice daily	25	0.05
	Total	483	1
**Dose Volume (ml/kg)**	0.2	6	0.02
	1	9	0.02
	2	3	0.01
	3	17	0.04
	5	201	0.51
	10	158	0.40
	Total	394	1
**Duration of dosing**	<1 d	33	0.07
	1 d	108	0.22
	3 d	36	0.07
	4 d	54	0.11
	5-6 d	25	0.05
	7-8 d	46	0.10
	14 d	48	0.10
	28 d	24	0.05
	30-31 d	63	0.13
	90-91 d	46	0.10
	Total	483	1
**Blood collection during in life study?**	NO	395	0.85
	YES	72	0.15
	Total	467	1
**Sacrifice method**	Anesthetic OD	32	0.07
	Cervical dislocation	12	0.02
	CO2 exposure	205	0.42
	Decapitation	70	0.14
	Exsanguinated	84	0.17

	Pentobarbital	80	0.17
	Total	483	1
**Fasted prior to sacrifice?**	NO	276	0.57
	YES	207	0.43
	Total	483	1
**Anesthetic use**	NO	186	0.39
	YES	297	0.61
	Total	483	1
**Interval between last dose and sacrifice (hrs)**	0	10	0.02
	3-6 h	74	0.16
	12	5	0.01
	24	346	0.77
	48	10	0.02
	168	4	0.01
	Total	449	1
**Time of day of Sacrifice**	AM	366	0.93
	PM	28	0.07
	Total	394	1
**Exsanguinated?**	NO	19	0.05
	YES	396	0.95
	Total	415	1
**Organ Sampled**	KIDNEY	124	0.26
	LIVER	359	0.74
	Total	483	1
**Organ Section**	edge of left lobe	21	0.04
	kidney cortex	16	0.03
	kidney medulla	16	0.03
	left & median lobes	12	0.03
	left lateral lobe	5	0.01
	left lobe	76	0.16
	left median lobe	69	0.14
	random	15	0.03
	right lateral lobe	4	0.01
	right median lobe	88	0.18
	whole kidney	92	0.19
	whole liver	64	0.13
	Total	478	1
**Method of Sample Preservation**	RNALater	39	0.08
	Snap Freeze	415	0.86
	Other	29	0.06
	Total	483	1
**RNA extraction protocol**	Qiagen RNeasy Kit	379	0.92
	Triazole	31	0.08
	Total	410	1
**Quality metrics**	260/280 nm	234	0.62
	28s/18s rRNA	119	0.32
	Both	24	0.06
	Total	377	1
**Sample prep protocol**	ENZO Kit	355	0.87
	IVT Labeling Kit	53	0.13
	Total	408	1
