# Supplementary material for: Sources of variation in baseline gene expression levels from toxicogenomics study control animals across multiple laboratories
Source: BMC Genomics. 2008 Jun 12;9:285. doi: 10.1186/1471-2164-9-285 (PMC2453529; doi:10.1186/1471-2164-9-285)
Supplement: Additional file 2 — Principal component analyses of tissue-array sets. Principal components were computed for normalized intensities from samples of given tissue-array combinations. Plots of the first two principal components are shown with the axis indicating the amount of variance in each component as a percentage of the total. [file 1471-2164-9-285-S2.ppt]

## Slide 1
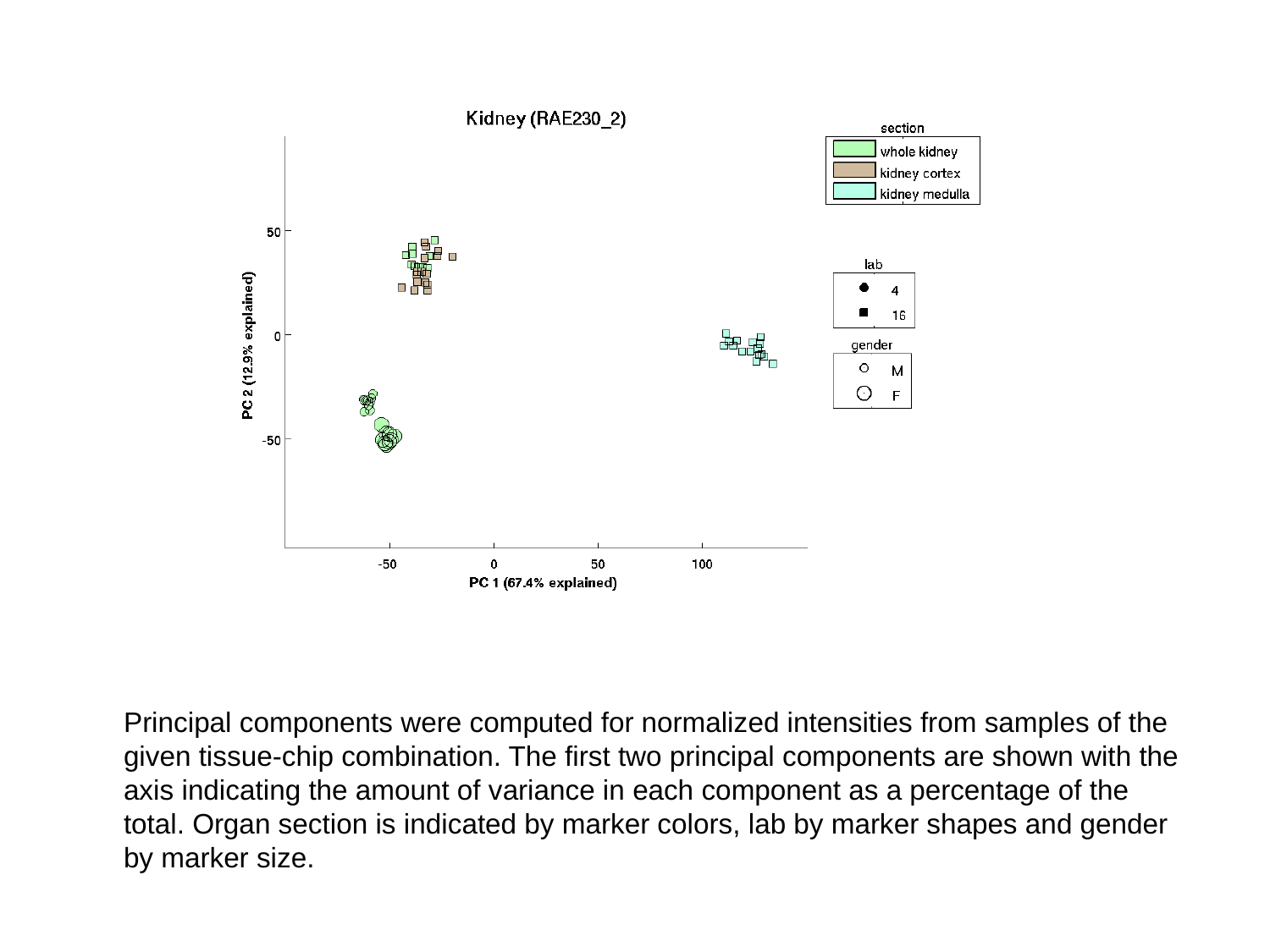

# Principal components were computed for normalized intensities from samples of the given tissue-chip combination. The first two principal components are shown with the axis indicating the amount of variance in each component as a percentage of the total. Organ section is indicated by marker colors, lab by marker shapes and gender by marker size.

## Slide 2
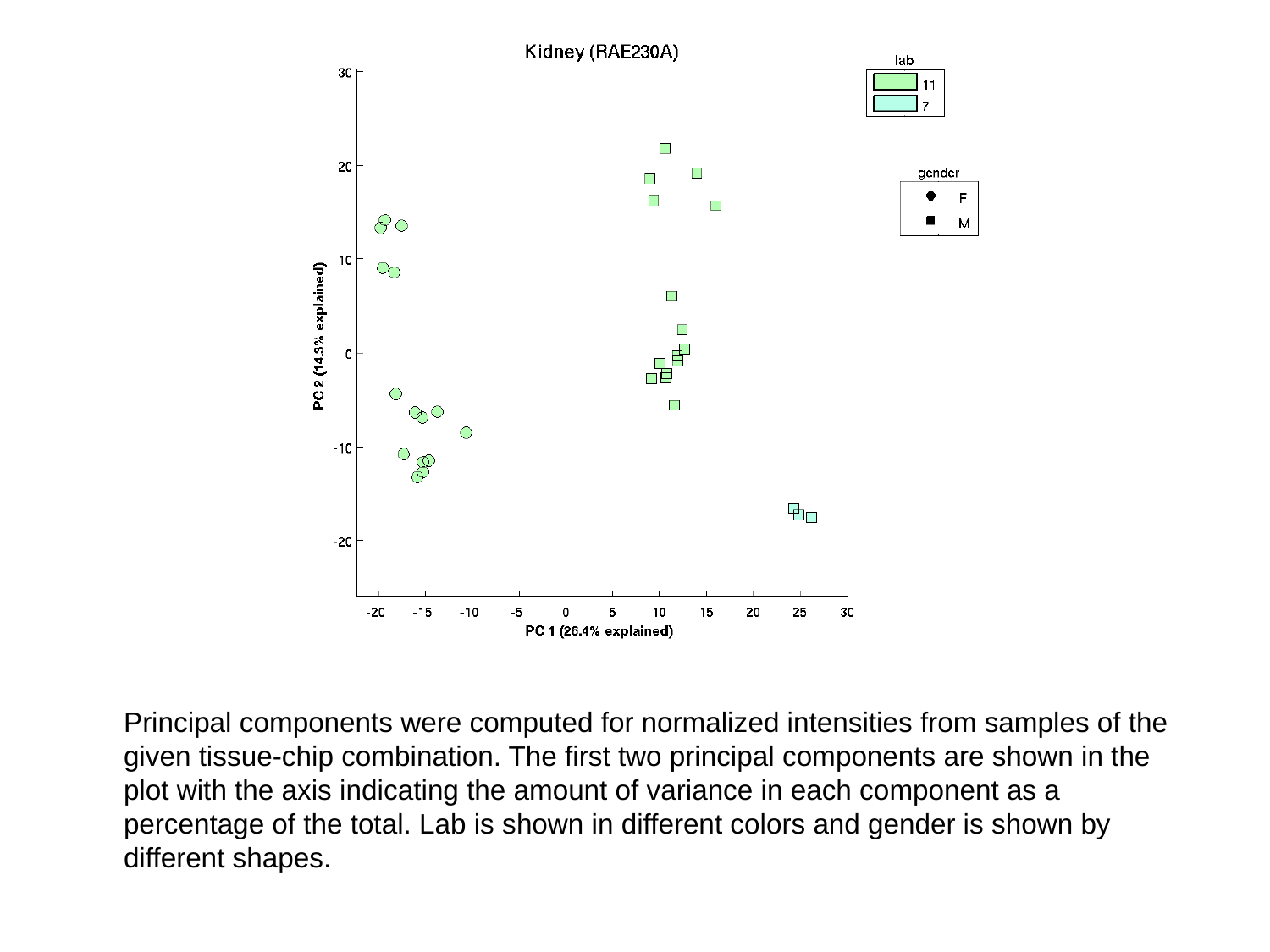

# Principal components were computed for normalized intensities from samples of the given tissue-chip combination. The first two principal components are shown in the plot with the axis indicating the amount of variance in each component as a percentage of the total. Lab is shown in different colors and gender is shown by different shapes.

## Slide 3
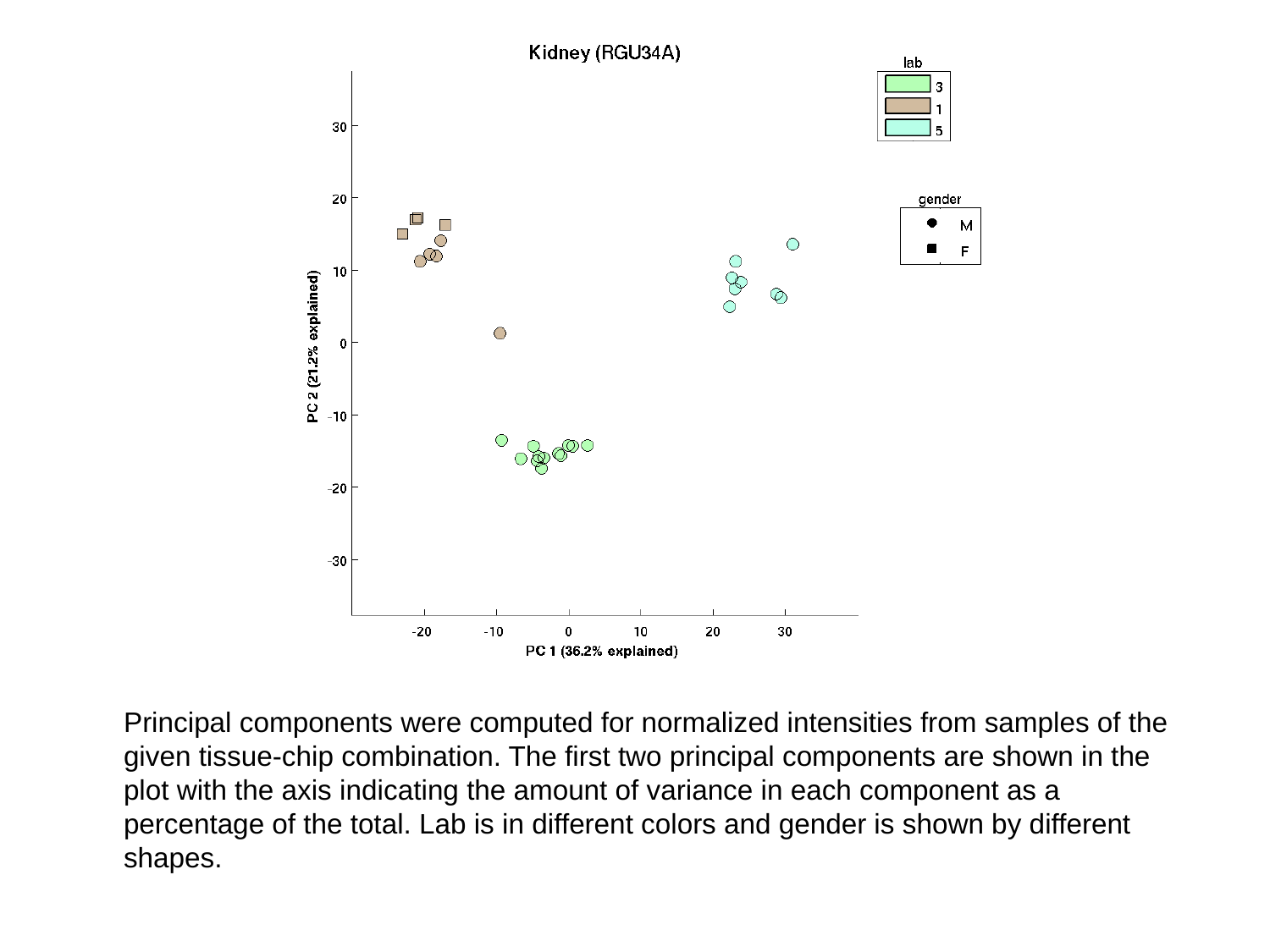

# Principal components were computed for normalized intensities from samples of the given tissue-chip combination. The first two principal components are shown in the plot with the axis indicating the amount of variance in each component as a percentage of the total. Lab is in different colors and gender is shown by different shapes.

## Slide 4
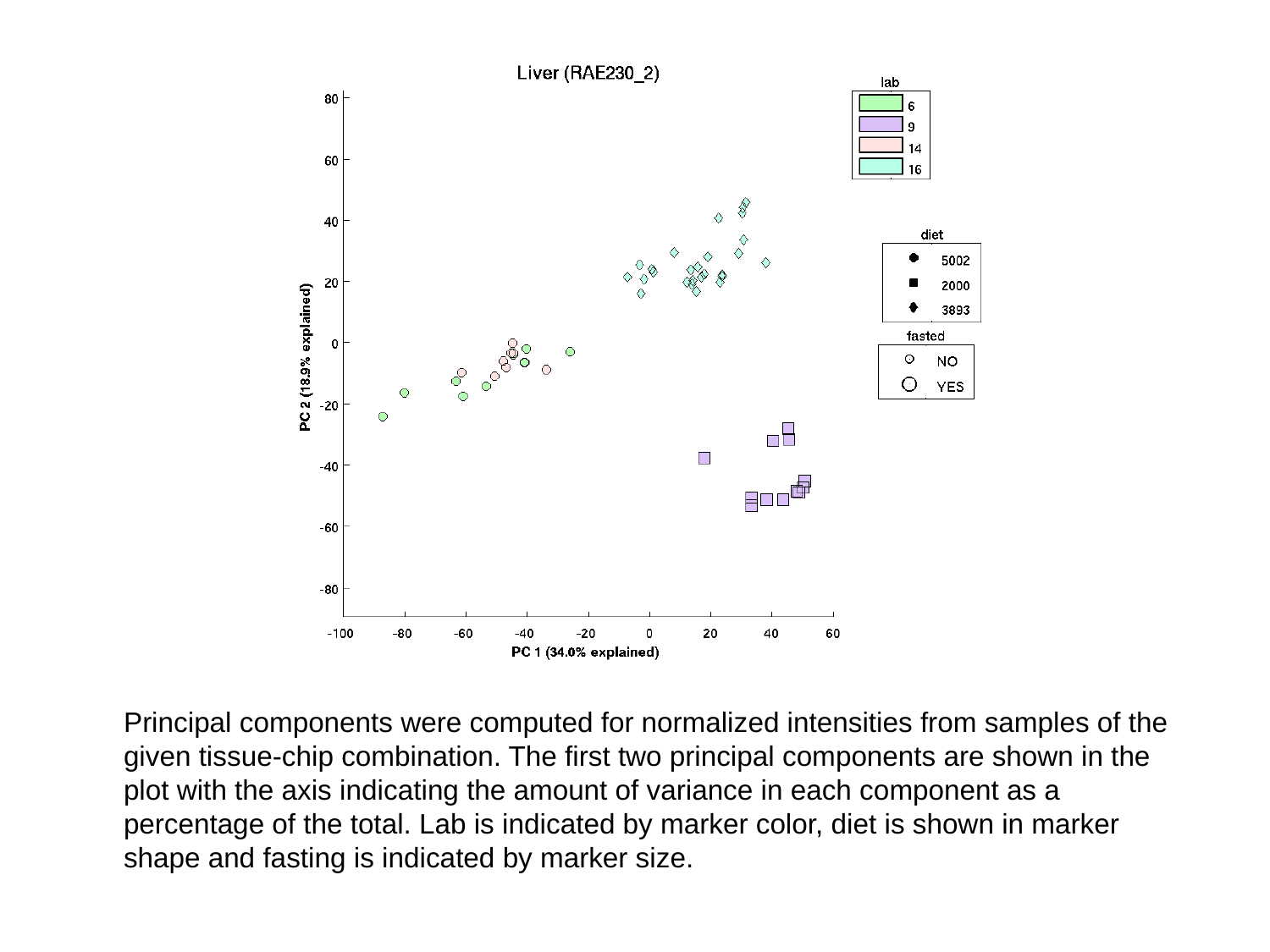

# Principal components were computed for normalized intensities from samples of the given tissue-chip combination. The first two principal components are shown in the plot with the axis indicating the amount of variance in each component as a percentage of the total. Lab is indicated by marker color, diet is shown in marker shape and fasting is indicated by marker size.

## Slide 5
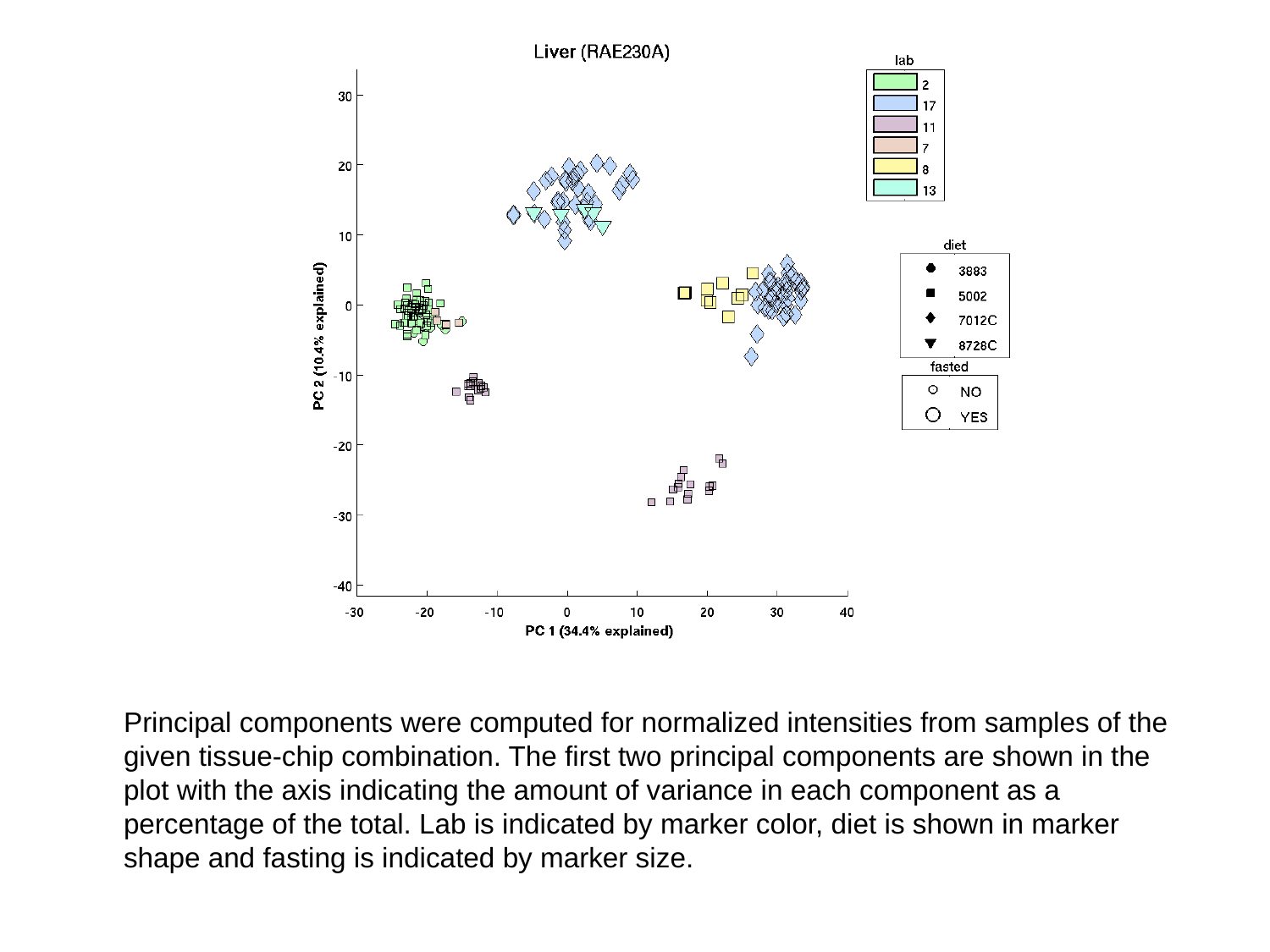

# Principal components were computed for normalized intensities from samples of the given tissue-chip combination. The first two principal components are shown in the plot with the axis indicating the amount of variance in each component as a percentage of the total. Lab is indicated by marker color, diet is shown in marker shape and fasting is indicated by marker size.

## Slide 6
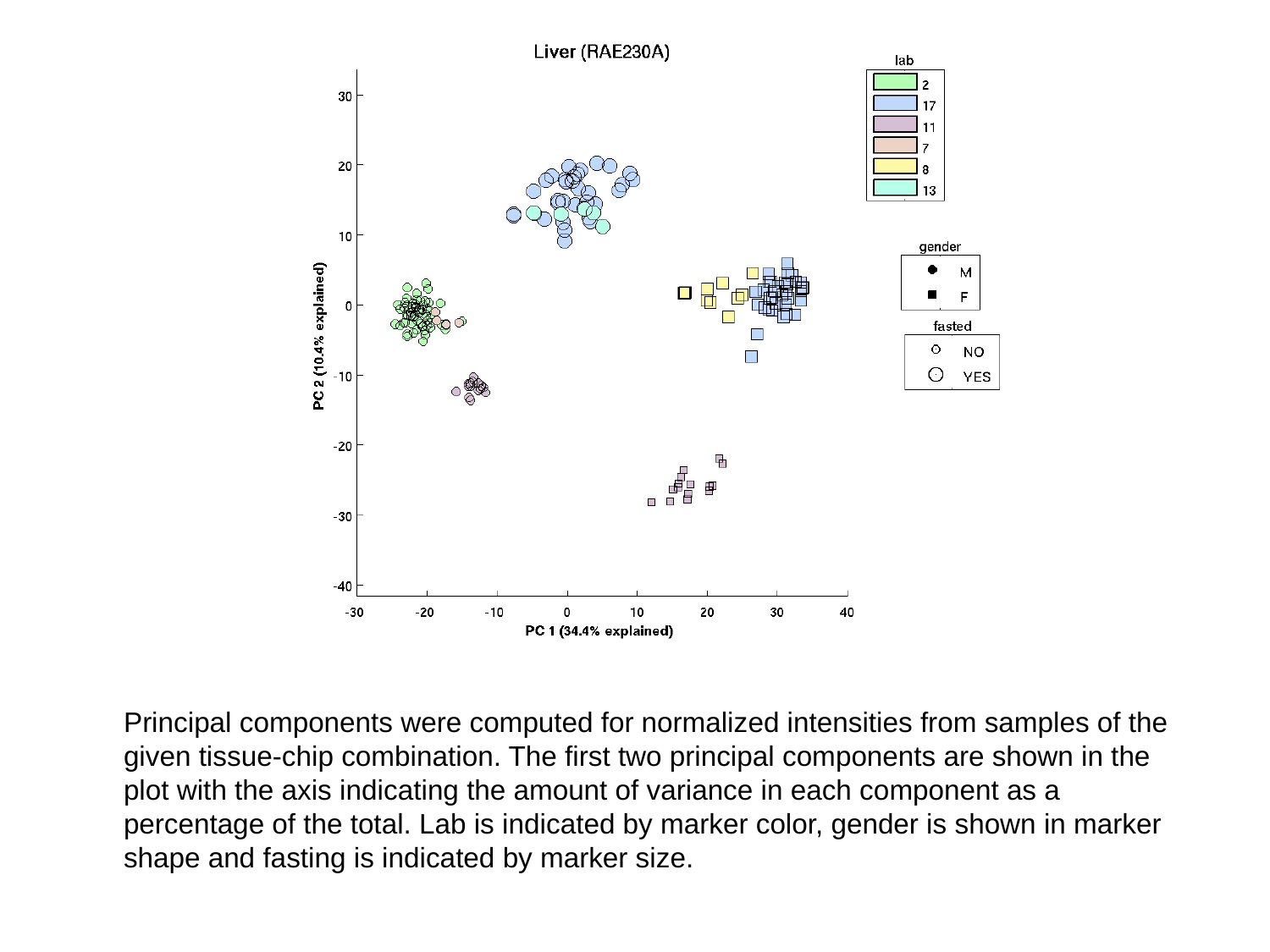

# Principal components were computed for normalized intensities from samples of the given tissue-chip combination. The first two principal components are shown in the plot with the axis indicating the amount of variance in each component as a percentage of the total. Lab is indicated by marker color, gender is shown in marker shape and fasting is indicated by marker size.

## Slide 7
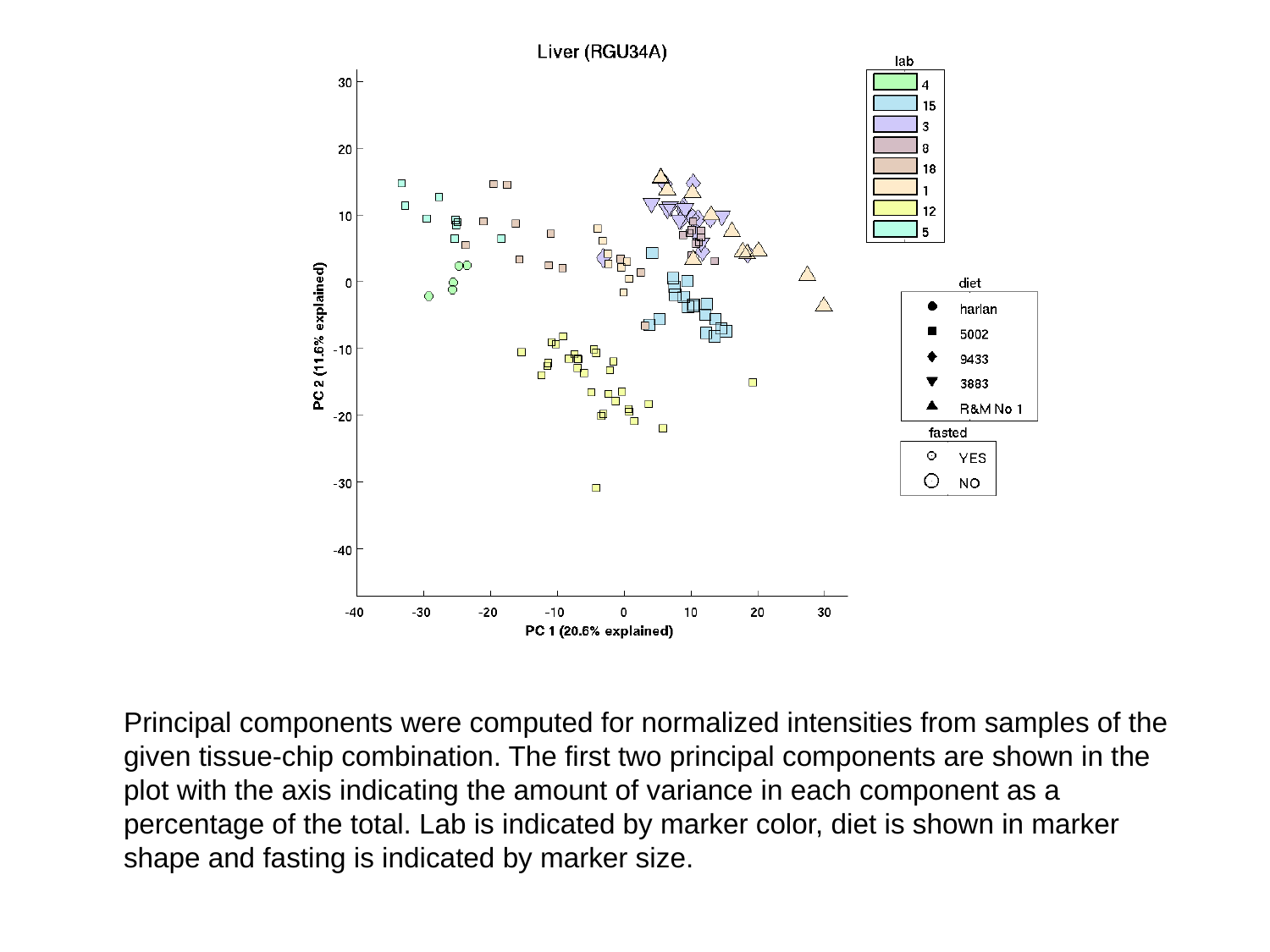

# Principal components were computed for normalized intensities from samples of the given tissue-chip combination. The first two principal components are shown in the plot with the axis indicating the amount of variance in each component as a percentage of the total. Lab is indicated by marker color, diet is shown in marker shape and fasting is indicated by marker size.

## Slide 8
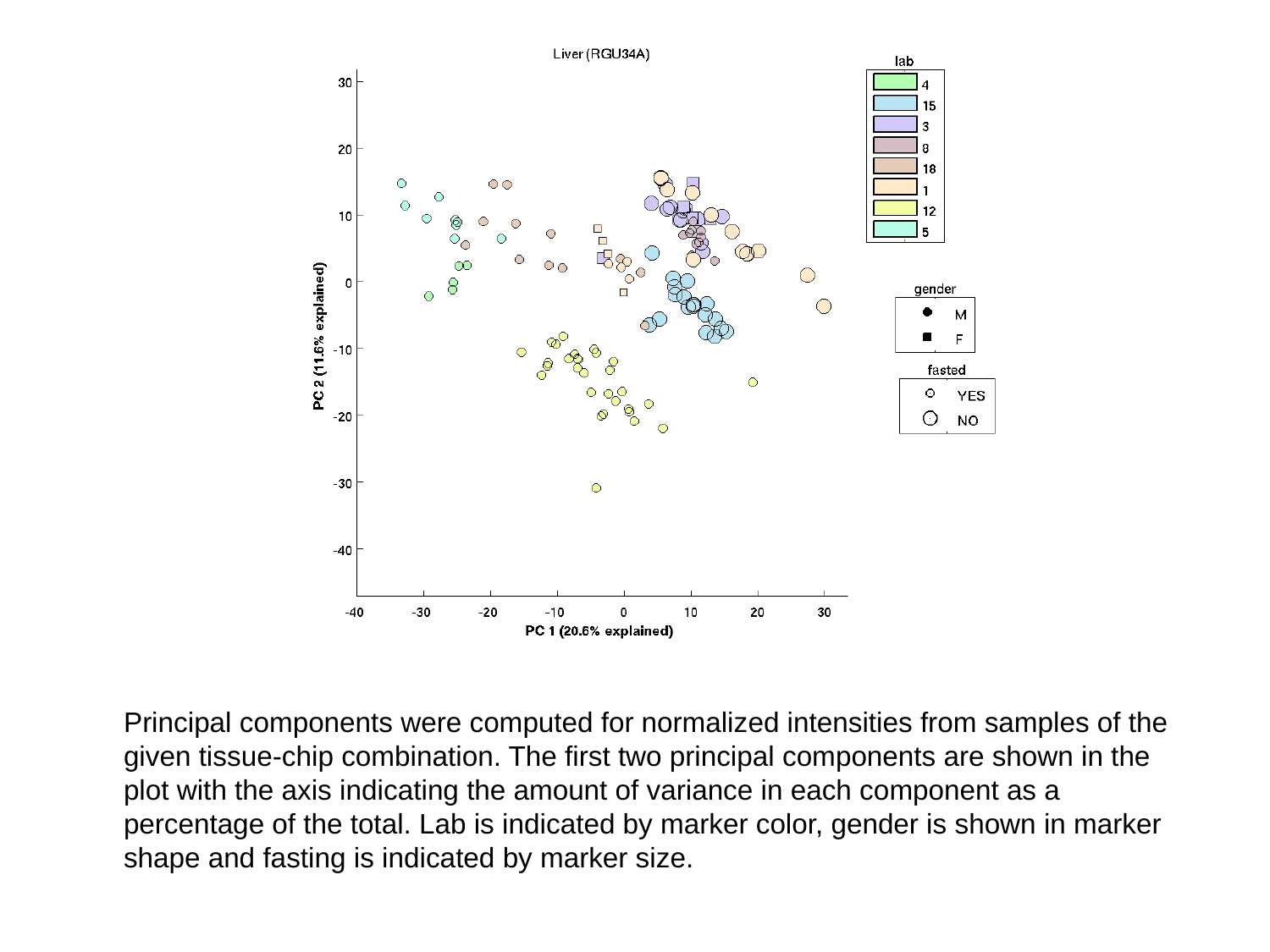

# Principal components were computed for normalized intensities from samples of the given tissue-chip combination. The first two principal components are shown in the plot with the axis indicating the amount of variance in each component as a percentage of the total. Lab is indicated by marker color, gender is shown in marker shape and fasting is indicated by marker size.
