# Supplementary figures and images for: Sources of variation in baseline gene expression levels from toxicogenomics study control animals across multiple laboratories
Source: BMC Genomics. 2008 Jun 12;9:285. doi: 10.1186/1471-2164-9-285 (PMC2453529; doi:10.1186/1471-2164-9-285)

## Slide 1
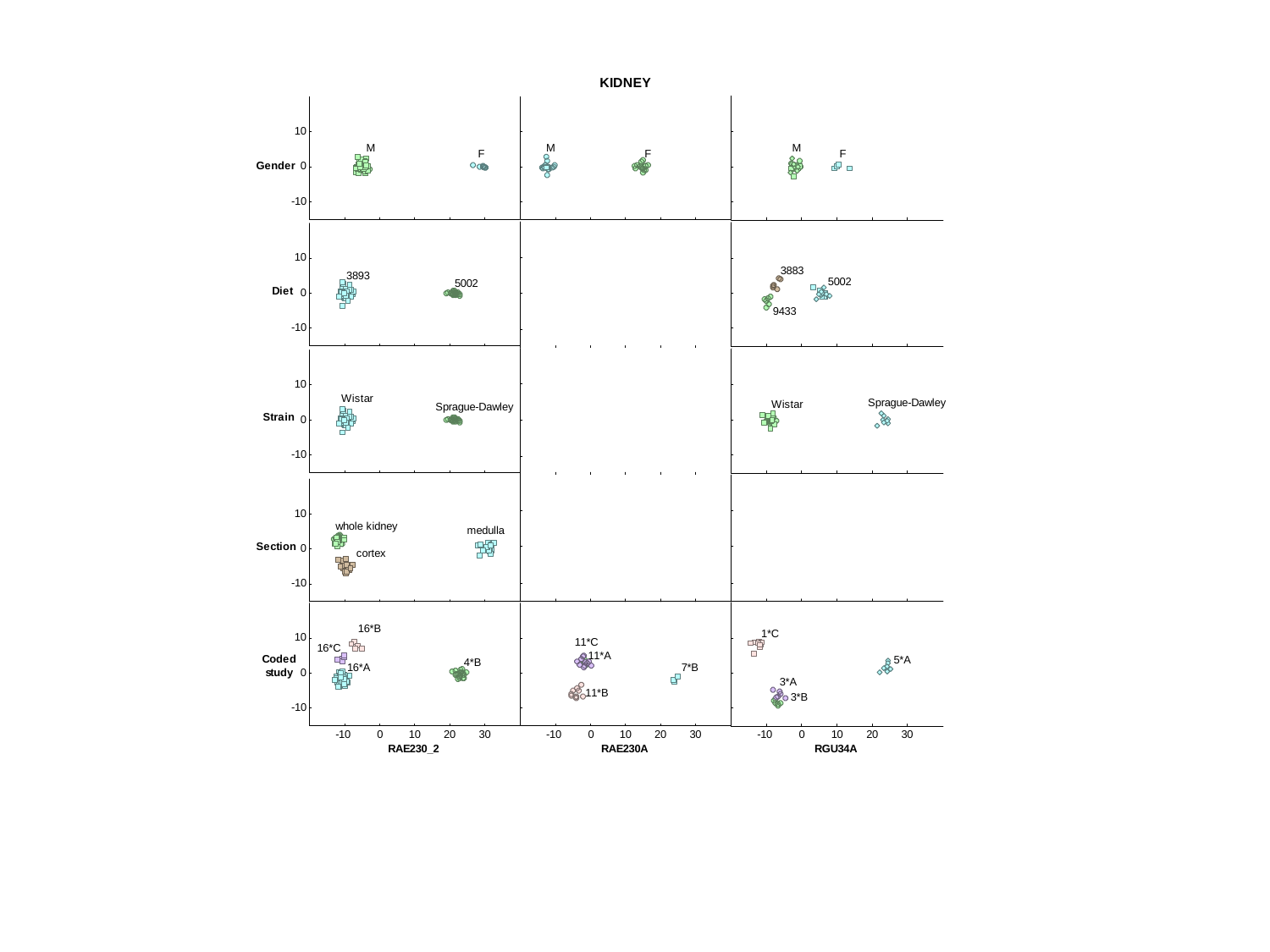

Supplement: Additional file 4 — Canonical variable plots of control kidney data. Each panel shows the first two canonical variables, which represent the maximum achievable separation for each factor (in rows) and array type (column). Each point in the plot represents an individual sample. The marker color along with text indicates the factor level for the sample and the shape indicates the site where the data were generated. [file 1471-2164-9-285-S4.ppt]
