# Supplementary material for: Sources of variation in baseline gene expression levels from toxicogenomics study control animals across multiple laboratories
Source: BMC Genomics. 2008 Jun 12;9:285. doi: 10.1186/1471-2164-9-285 (PMC2453529; doi:10.1186/1471-2164-9-285)
Supplement: Additional file 5 — Confounding relationships for selected factors in kidney. This table shows factors observed to be confounded with those analyzed in Figure 2. The confounding relationships were determined by fitting a partition tree model to each factor (gender, diet, strain, or fasted), using the other 16 factors as predictors. [file 1471-2164-9-285-S5.doc]

**Additional Table 3: Confounding relationships for selected factors in kidney.**

|  | *RAE230A* | *RAE230 2.0* | *RGU34A* |
| --- | --- | --- | --- |
| Gender | None | None | None |
| Diet | n/a – same diet for all | Strain | RNAAmount + Route / Vehicle |
| Strain | n/a – all Sprague-Dawley | Fasted / SacMethod / Anesthetic / Diet | Age / Fixation / Vehicle / RNAAmount |
| Fasted | n/a – all No | Strain / SacMethod / Anesthetic / Diet | RNAAmount / SacMethod / Age / Diet |
