# Supplementary material for: Sources of variation in baseline gene expression levels from toxicogenomics study control animals across multiple laboratories
Source: BMC Genomics. 2008 Jun 12;9:285. doi: 10.1186/1471-2164-9-285 (PMC2453529; doi:10.1186/1471-2164-9-285)
Supplement: Additional file 8 — Sources of data used to identify gender-specific and fasting-related genes. The institution number, study code, and key descriptors for the data sets used in the EPIG analyses of genes associated with gender-selective expression or with expression changed by fasting state are listed. Study codes were assigned to distinguish independent studies submitted from the same institution. [file 1471-2164-9-285-S8.doc]

Source of data used to identify gender-specific genes.

| Institution | Study code | Sex | Tissue | Number of animals | Strain | Chip type | Fasted? |
| --- | --- | --- | --- | --- | --- | --- | --- |
| 1 | B | M | Liver | 10 | Wistar | U34A | No |
| 1 | C | F | Liver | 4 | Wistar | U34A | No |
| 1 | C | F | Kidney | 4 | Wistar | U34A | Yes |
| 1 | C | M | Liver | 4 | Wistar | U34A | No |
| 1 | C | M | Kidney | 5 | Wistar | U34A | Yes |
| 3 | A | M | Liver | 3 | Wistar | U34A | No |
| 3 | C | F | Liver | 6 | Wistar | U34A | No |
| 3 | C | M | Liver | 6 | Wistar | U34A | No |
| 3 | D | M | Liver | 4 | Wistar | U34A | No |
| 4 | B | F | Kidney | 9 | SD | RAE230_2 | No |
| 4 | B | M | Kidney | 10 | SD | RAE230_2 | No |
| 11 | A | F | Liver and Kidney | 5 | SD | 230A | No |
| 11 | A | M | Liver and Kidney | 5 | SD | 230A | No |
| 11 | B | F | Liver and Kidney | 5 | SD | 230A | No |
| 11 | B | M | Liver and Kidney | 5 | SD | 230A | No |
| 11 | C | F | Liver and Kidney | 5 | SD | 230A | No |
| 11 | C | M | Liver and Kidney | 5 | SD | 230A | No |
| 17 | A | F | Liver | 15 | SD | 230A | No |
| 17 | D | F | Liver | 4 | SD | 230A | No |
| 17 | E | F | Liver | 14 | SD | 230A | No |
| 17 | F | M | Liver | 16 | SD | 230A | No |
| 17 | G | M | Liver | 18 | SD | 230A | No |

SD, Sprague-Dawley

Source of data used to identify fasting genes.

| Institution | Study code | Sex | Strain | Number of animals | Chip type | Fasted? |
| --- | --- | --- | --- | --- | --- | --- |
| 2 | A | M | Wistar | 12 | 230A | No |
| 2 | D | M | Wistar | 12 | 230A | No |
| 2 | E | M | Wistar | 12 | 230A | No |
| 7 | A | M | SD | 5 | 230A | No |
| 8 | B | F | Fisher | 11 | 230A | Yes |
| 11 | A | F | SD | 5 | 230A | No |
| 11 | A | M | SD | 5 | 230A | No |
| 11 | B | F | SD | 5 | 230A | No |
| 11 | B | M | SD | 5 | 230A | No |
| 17 | C | F | SD | 5 | 230A | Yes |
| 17 | D | F | SD | 4 | 230A | Yes |
| 17 | F | M | SD | 16 | 230A | Yes |
| 17 | G | M | SD | 18 | 230A | Yes |

SD, Sprague-Dawley
