# Supplementary material for: Sources of variation in baseline gene expression levels from toxicogenomics study control animals across multiple laboratories
Source: BMC Genomics. 2008 Jun 12;9:285. doi: 10.1186/1471-2164-9-285 (PMC2453529; doi:10.1186/1471-2164-9-285)
Supplement: Additional file 10 — Results of real-time RT-PCR analysis of gender-difference genes in livers and kidneys of F344/N rats. Gender-selective expression of a subset of genes identified by EPIG analysis of the control animal dataset was verified in an independent study using RT-PCR. Each RT-PCR experiment was run twice with similar results. The expression levels in each animal were evaluated in duplicate, with 3 animals per group. [file 1471-2164-9-285-S10.doc]

Real-time RT-PCR Analysis of Gender-difference Genes in Livers and Kidneys of F344/N Rats

Liver Liver M/F Kidney Kidney M/F

Gene categories Male Female (fold) Male Female (fold)

# Category #1

*X76456, Afm* 1.0 ± 0.1 0.5 ± 0.1 1.8* 1.0 ± 0.2 0.6 ± 0.1 1.4

*NM_013215, Akr7a3* 1.0 ± 0.2 0.6 ± 0.1 1.6 1.0 ± 0.2 0.7 ± 0.1 1.5

*NM_031700, Cldn3* 1.0 ± 0.1 0.5 ± 0.1 2.0* 1.0 ± 0.2 1.4 ± 0.1 -1.4

*NM_031546, Rgn* 1.0 ± 0.1 0.8 ± 0.1 1.2 1.0 ± 0.3 0.1 ± 0.0 9.0*

*NM_017166, Stmn1* 1.0 ± 0.2 0.4 ± 0.1 2.4* 1.0 ± 0.1 0.9 ± 0.1 1.0

# Category #2

*NM_053781, Akr1b7* 1.0 ± 0.5 267± 12 -267* 1.0 ± 0.2 1.4 ± 0.1 -1.4

*AI598546, Eif2s3x* 1.0 ± 0.4 2.0 ± 0.2 -2.1* 1.0 ± 0.4 2.1 ± 0.8 -2.1

*NM_022521, Oat* 1.0 ± 0.1 2.0 ± 0.2 -2.0* 1.0 ± 0.1 3.7 ± 0.4 -3.7*

*AA945082, Yc2*  1.0 ± 0.2 13 ±1.6 -13* 1.0 ± 0.1 1.9 ± 0.2 1.9*

# Category #3

*U09472,Cyp3a11* 1.0 ± 0.1 0.0 ± 0.0 300* 1.0 ± 0.2 3.7 ± 0.1 -3.7*

*NM_012552, Ela1* 1.0 ± 0.1 0.3 ± 0.1 3.3* 1.0 ± 0.1 1.0 ± 0.1 1.0

*AA892297, Hdac2* 1.0 ± 0.4 0.7 ± 0.0 1.4 1.0 ± 0.0 1.1 ± 0.2 1.1

*M27882, Spink1* 1.0 ± 0.1 0.0 ±0.0 94* 1.0 ± 0.2 2.1 ± 0.5 -2.1

# Category #4

*AJ302031, A1bg* 1.0 ± 0.4 274 ± 60 -274* 1.0 ± 0.5 0.3 ± 0.1 3.3

*NM_031753, Alcam* 1.0 ± 0.1 2.1 ± 0.3 -2.1* 1.0 ± 0.2 1.5 ± 0.2 -1.5

*NM_031561, Cd36* 1.0 ± 0.1 12 ± 3.5 -12* 1.0 ± 0.2 1.4 ± 0.4 -1.4

*NM_031572, Cyp2c40* 1.0 ± 0.2 124 ±0.0 -124* 1.0 ± 0.5 0.8 ± 0.4 1.2

*NM_017126, Fdx1*  1.0 ± 0.1 3.3 ± 0.4 -3.4* 1.0 ± 0.8 1.0 ± 0.2 1.0

# Category #5

*BM383010, Akr1c12* 1.0 ± 0.1 0.7 ± 0.0 -1.4 1.0 ± 0.1 0.1 ± 0.0 13.*

*NM_031565, Ces1* 1.0 ± 0.2 1.4 ± 0.2 -1.4 1.0 ± 0.1 0.3 ± 0.1 3.3*

*NM_031324, Prep* 1.0 ± 0.1 1.2 ± 0.2 -1.2 1.0 ± 0.1 0.7 ± 0.2 -1.4

*NM_031584, Slc22a2*  1.0 ± 0.5 0.3 ± 0.1 3.5* 1.0 ± 0.1 0.1 ± 0.0 9.0*

# Category #6

*BI275292,Angpt2* 1.0 ± 0.2 1.3 ± 0.2 -1.3 1.0 ± 0.2 7.3 ± 1.2 -7.3*

*M29853, Cyp4b1* 1.0 ± 0.1 1.2 ± 0.2 -1.2 1.0 ± 0.1 5.2 ± 1.3 -5.2*

*NM_031514, Jak2* 1.0 ± 0.2 1.7 ± 0.3 -1.7* 1.0 ± 0.3 4.1 ± 0.3 -4.1*

*NM_053537, Slc22a7*  1.0 ± 0.2 1.1 ± 0.2 -1.1 1.0 ± 0.2 8.7 ± 0.5 -8.7*

# Category #7

*X95189, Acox2* 1.0 ± 0.2 1.0 ± 0.0 1.0 1.0 ± 0.3 1.7 ± 0.1 -1.7

*L46791, Ces3*  1.0 ± 0.1 0.5 ± 0.1 2.2* 1.0 ± 0.1 3.5 ± 0.5 -3.5*

*AI013390, Cited2* 1.0 ± 0.1 0.5 ± 0.0 2.0* 1.0 ± 0.2 0.6 ± 0.1 1.6

*J02844, Crot* 1.0 ± 0.1 1.6 ± 0.3 -1.6 1.0 ± 0.1 2.0 ± 0.2 -2.0*

# Category #8

*AI104324,Dnaja4*  1.0 ± 0.1 0.9 ± 0.1 1.1 1.0 ± 0.0 0.9 ± 0.1 1.1

*NM_012630, Prlr*  1.0 ± 0.1 109 ± 12 -109* 1.0 ± 0.1 0.1 ± 0.0 7.2*

*NM_021751,Prom1* 1.0 ± 0.2 3.5 ± 0.7 -3.5* 1.0 ± 0.2 0.4 ± 0.0 2.3*

*NM_019269, Slc22a5* 1.0 ± 0.2 1.5 ± 0.3 -1.5* 1.0 ± 0.1 0.5 ± 0.1 2.0*

Data are mean ± SEM of 3. *Significant sex difference p<0.05.
