# Supplementary material for: Sources of variation in baseline gene expression levels from toxicogenomics study control animals across multiple laboratories
Source: BMC Genomics. 2008 Jun 12;9:285. doi: 10.1186/1471-2164-9-285 (PMC2453529; doi:10.1186/1471-2164-9-285)
Supplement: Additional file 11 — Gene ontology analysis of gender-specific genes in the rat liver and kidney. Results of analysis performed using the Gene Ontology function of GeneGo with the help of KEGG [31]. The max(-log(p-value)) is the result of a hypergeometric test using GeneGo. The higher the score, the greater the significance of the network. Network objects refers to the number of objects identified divided by the total number of objects in the network in the GeneGo database. [file 1471-2164-9-285-S11.doc]

Gene Ontology analysis of gender-specific genes in the rat liver.

| **Processes Name** | **Gene Abbreviations** | **max(-log(pValue))** | **Network objects** |
| --- | --- | --- | --- |
| xenobiotic metabolism | AHR,CYP2C9,CYP3A4,GSTM1,CYP3A43,CYP3A7,CYP3A3 | 17.272 | 7/31 |
| response to xenobiotic stimulus | AHR,CYP2C9,CYP3A4,GSTM1,CYP3A43,CYP3A7,CYP3A3 | 16.369 | 7/35 |
| cellular lipid metabolism | Acyl-CoA synthetase,HSD17B2,AKR1C3,HSD11B1,CD36,FDX1,Prolactin receptor,SULT1E1,CES1,PP2A structural,GPAM,SULT2A1,PI3K class II,ACSL5,HAOX2,S5AR,PPP2R1B,NUDT4 | 13.961 | 18/436 |
| lipid metabolism | Acyl-CoA synthetase,HSD17B2,AKR1C3,HSD11B1,CD36,FDX1,CYP3A4,Prolactin receptor,SULT1E1,CES1,PP2A structural,GPAM,SULT2A1,PI3K class II,ACSL5,HAOX2,S5AR,PPP2R1B,NUDT4 | 12.533 | 19/532 |
| steroid metabolism | HSD17B2,AKR1C3,HSD11B1,FDX1,Prolactin receptor,SULT1E1,SULT2A1,S5AR | 9.517 | 8/128 |
| substrate-bound cell migration, cell contraction | SUR,SUR2 | 9.043 | 2/2 |
| vacuolar protein catabolism | Carboxypeptidase A (pancreatic),CPA2 | 9.043 | 2/2 |
| hormone metabolism | HSD17B2,AKR1C3,HSD11B1,SULT1E1,PACE4,S5AR | 8.056 | 6/85 |
| positive regulation of osteoclast differentiation | TIEG,TIEG1 | 7.265 | 2/4 |
| fatty acid metabolism | Acyl-CoA synthetase,AKR1C3,CD36,CES1,GPAM,ACSL5,HAOX2 | 7.022 | 7/142 |
| carboxylic acid metabolism | Acyl-CoA synthetase,AKR1C3,CD36,SLC7A7,CES1,PP2A structural,GPAM,SLC7A5,CSAD,ACSL5,HAOX2,PPP2R1B | 7.019 | 12/383 |
| organic acid metabolism | Acyl-CoA synthetase,AKR1C3,CD36,SLC7A7,CES1,PP2A structural,GPAM,SLC7A5,CSAD,ACSL5,HAOX2,PPP2R1B | 6.973 | 12/385 |
| positive regulation of monocyte differentiation | TIEG,TIEG1 | 6.363 | 2/6 |
| negative regulation of peptidyl-tyrosine phosphorylation | PP2A structural,PPP2R1B | 6.034 | 2/7 |
| negative regulation of tyrosine phosphorylation of Stat3 protein | PP2A structural,PPP2R1B | 6.034 | 2/7 |
| negative regulation of JAK-STAT cascade | PP2A structural,PPP2R1B | 6.034 | 2/7 |
| negative regulation of tyrosine phosphorylation of STAT protein | PP2A structural,PPP2R1B | 6.034 | 2/7 |
| regulation of cell adhesion | KNG,Prolactin receptor,PP2A structural,PPP2R1B | 6.021 | 4/52 |
| protein amino acid dephosphorylation | PTPD1,PP2A regulatory,PPP2R2A,PP2A structural,PPP2R1B | 5.870 | 5/89 |
| regulation of development | Prolactin receptor,TIEG,Calpactin I light chain,TIEG1,Reticulon 4,PP2A structural,IBP,RING6,PPP2R1B, | 5.728 | 9/278 |
| regulation of cell differentiation | Prolactin receptor,TIEG,Calpactin I light chain,TIEG1,PP2A structural,IBP,RING6,PPP2R1B | 5.661 | 8/229 |
| bone mineralization | TIEG,TIEG1,Osteomodulin | 5.485 | 3/30 |
| dephosphorylation | PTPD1,PP2A regulatory,PPP2R2A,PP2A structural,PPP2R1B | 5.454 | 5/98 |
| biological process unknown | Olfactory receptor,Glycoprotein Ib-alpha,PI3K class II (CII-gamma),TCTEX,VAMP7,H-rev 107 protein,PI3K class II,ABHD2,DYNLT3 | 5.426 | 9/291 |
| response to chemical stimulus | AHR,Carbonic anhydrase III,CYP2C9,CYP3A4,GSTM1,CYP3A43,CES1,CYP3A7,CYP3A3,PP2A structural,ENPP2,PPP2R1B | 5.277 | 12/472 |

Gene Ontology analysis of gender-specific genes in the rat kidney.

| Processes Name | Gene Names | | | max(-log(pValue)) | | Network objects |
| --- | --- | --- | --- | --- | --- | --- |
| lipid metabolism | PLD1,Angiotensinogen,Myosin Va,P-glycoprotein,HSD17B2,HSD11B1,ADHG,Alpha-synuclein,LPL,HPGD,IDI1,FDFT1,ACADM,Prolactinreceptor,CES1,PGHD,RBP1,Non-A beta component of AD amyloid,Alpha-synuclein filaments,AZGP1,Retinol-binding protein,LTB4DH,CROT,ACOX2,AAAD,SULT2B1, | | | 13.953 | | 26/532 |
| cellular lipid metabolism | PLD1,Angiotensinogen,Myosin Va,HSD17B2,HSD11B1,ADHG,Alpha-synuclein,LPL,HPGD,IDI1,FDFT1,ACADM,Prolactin receptor,CES1,PGHD,RBP1,Non-A beta component of AD amyloid,Alpha-synuclein filaments,Retinol-binding protein,LTB4DH,CROT,ACOX2,SULT2B1 | | | 13.711 | | 23/436 |
| aromatic compound metabolism | Occludin,FTCD,Alpha-synuclein,CYP1A1,HPD,Non-A beta component of AD amyloid,Alpha-synuclein filaments, GAMT,FAAA | | | 13.654 | | 9/64 |
| carboxylic acid metabolism | Angiotensinogen,Myosin Va,ACK1,FTCD,LPL,HPGD,ACADM,CES1,ASNS,PGHD,GPD2,HPD,OAT,LTB4DH,CROT,CSAD,ACOX2,GAMT,FAAA | | | 10.596 | | 19/383 |
| organic acid metabolism | Angiotensinogen,MyosinVa,ACK1,FTCD,LPL, HPGD,ACADM,CES1,ASNS,PGHD,GPD2, HPD,OAT,LTB4DH,CROT,CSAD,ACOX2,GAMT,FAAA | | | 10.523 | | 19/385 |
| vitamin transport | Cubilin,SLC19A1,VDB,SMVT | | | 10.231 | | 4/12 |
| response to drug | MDR1,P-glycoprotein,Alpha-synuclein,Non-A beta component of AD amyloid,Alpha-synuclein filaments,GSTA1 | | | 9.299 | | 6/44 |
| positive regulation of neurotransmitter secretion | AAlpha-synuclein,Non-A beta component of AD amyloid,Alpha-synuclein filaments | | | 9.278 | | 3/6 |
| fatty acid metabolism | Angiotensinogen,Myosin Va,LPL,HPGD,ACADM,CES1,PGHD,LTB4DH,CROT,ACOX2, | | | 8.818 | | 10/142 |
| amino acid derivative biosynthesis | Alpha-synuclein,Non-A beta component of AD amyloid,Alpha-synuclein filaments,CSAD,GAMT | | | 8.436 | | 5/33 |
| regulation of NK T cell activation | CD1,CD1d, | | | 8.151 | | 2/2 |
| positive regulation of NK T cell activation | CD1,CD1d, | | | 8.151 | | 2/2 |
| antigen presentation, endogenous lipid antigen | CD1,CD1d, | | | 8.151 | | 2/2 |
| NK T cell activation | CD1,CD1d, | | | 8.151 | | 2/2 |
| positive regulation of T cell mediated cytotoxicity | CD1,CD1d, | | | 8.151 | | 2/2 |
| synaptic transmission, dopaminergic | Alpha-synuclein,Non-A beta component of AD amyloid,Alpha-synuclein filaments, | | | 7.881 | | 3/9 |
| isoprenoid metabolism | ADHG,IDI1,RBP1,Retinol-binding protein | | | 7.858 | | 4/21 |
| recombinational repair | Histone H2,Histone H2A,Nucleosome | | | 7.537 | | 3/10 |
| dopamine biosynthesis | Alpha-synuclein,Non-A beta component of AD amyloid,Alpha-synuclein filaments | | | 7.537 | | 3/10 |
| receptor guanylyl cyclase signaling pathway | G-protein gamma,Guanylate cyclase,GNG7 | | | 7.537 | | 3/10 |
| double-strand break repair via homologous recombination | Histone H2,Histone H2A,Nucleosome | | | 7.537 | | 3/10 |
| cellular nerve ensheathment | Fyn,Myosin Va,POU class III,Claudin 1 | | | 7.162 | | 4/25 |
| ionic insulation of neurons by glial cells | Fyn,Myosin Va,POU class III,Claudin 1 | | | 7.162 | | 4/25 |
| myelination | Fyn,Myosin Va,POU class III,Claudin 1 | | | 7.162 | | 4/25 |
| regulation of T cell mediated cytotoxicity | CD1,CD1d, | | | 7.063 | | 2/3 |
|  | |  |  | |  | |
|  | |  |  | |  | |
|  | |  |  | |  | |
|  | |  |  | |  | |
|  | |  |  | |  | |
|  | |  |  | |  | |
|  | |  |  | |  | |
|  | |  |  | |  | |
|  | |  |  | |  | |
