# Supplementary material for: Sources of variation in baseline gene expression levels from toxicogenomics study control animals across multiple laboratories
Source: BMC Genomics. 2008 Jun 12;9:285. doi: 10.1186/1471-2164-9-285 (PMC2453529; doi:10.1186/1471-2164-9-285)
Supplement: Additional file 13 — Gene ontology analysis of fasting-associated genes in the rat liver identified using a t-test or EPIG. Results of analysis performed using the Gene Ontology function of GeneGo with the help of KEGG [31] is the result of a hypergeometric test using GeneGo. The higher the score, the greater the significance of the network. Network objects refers to the number of objects identified divided by the total number of objects in the network in the GeneGo database. [file 1471-2164-9-285-S13.doc]

Gene Ontology analysis of fasting genes in the rat liver identified using t-test.

| Process Name | Gene Name | max(-log(pValue)) | Network objects |
| --- | --- | --- | --- |
| lipid metabolism | CYP2J2,PLA2,SCD,ACLY,HMDH,HMGCS1,Galpha(q)-specific peptide GPCRs,SREBP1,Acyl-CoA synthetase,MIF,CYP4F2,P-glycoprotein,MDR3,Fatty acid-binding protein,CYP17,CPT-1A,Beta-ketothiolase,Acetyl-CoA acyltransferase,IDI1,FDFT1,ERG1,LSS,DHCR7,CYP51A1,SC4MOL,I-FABP,AFP,CRAT,B-FABP,CPT II,S14 protein,CYP4A11,APOA5,HADHB,ACSL3,PAHX,E-FABP,NSDHL,Monoglyceride lipase,GPAM,ACAT2,RDH11,PLA2G12,DCI,AFP-cyclopeptide,DHC24,ACAA2,ACSL5,PTE2,PLCC,CYP4F11,CTE1,AAAD,PXR,PMVK,HPLC2 | 56.303 | 56/532 |
| cellular lipid metabolism | CYP2J2,PLA2,SCD,HMDH,Galpha(q)-specific peptide GPCRs,SREBP1,Acyl-CoA synthetase,MIF,CYP4F2, Fatty acid-binding protein,CYP17,CPT-1A,Beta- ketothiolase,Acetyl-CoA acyltransferase,IDI1,FDFT1,ERG1,LSS,DHCR7,CYP51A1,SC4MOL,I-FABP,AFP,CRAT,B-FABP,CPT II,CYP4A11,APOA5,HADHB,ACSL3,PAHX,E-FABP,NSDHL,GPAM,RDH11,DCI,AFP-cyclopeptide,DHC24,ACAA2,ACSL5,PTE2,PLCC,CYP4F11,CTE1,PXR,HPLC2 | 45.614 | 46/436 |
| fatty acid metabolism | CYP2J2,PLA2,SCD,Acyl-CoA synthetase,MIF,CYP4F2,Fatty acid-binding protein,CPT-1A,Beta- ketothiolase,Acetyl-CoA acyltransferase,SC4MOL,I-FABP,CRAT,B-FABP,CPT II,CYP4A11, HADHB,ACSL3,PAHX,GPAM,DCI,ACSL5,PTE2,CYP4F11,CTE1,HPLC2 | 39.014 | 26/142 |
| carboxylic acid metabolism | CYP2J2,PLA2,SCD,ACLY,Acyl-CoA synthetase,MIF,CYP4F2,Fatty acid-binding protein,CPT-1A,Beta- ketothiolase,Acetyl-CoA acyltransferase,ME1,SC4MOL,I-FABP,CRAT,ARLY,B-FABP,CPT II,CYP4A11,HADHB,ACSL3,BCKD-E1beta,PAHX,BUP1,DHPR,OTC,GPAM,DCI,PSAT, SSDH,ACSL5,PTE2,CYP4F11,GCSP,CTE1,HPLC2 | 31.667 | 36/383 |
| organic acid metabolism | CYP2J2,PLA2,SCD,ACLY,Acyl-CoA synthetase,MIF,CYP4F2,Fatty acid-binding protein,CPT-1A,Beta- ketothiolase,Acetyl-CoA acyltransferase,ME1,SC4MOL,I-FABP,CRAT,ARLY,B-FABP,CPT II,CYP4A11,HADHB, ACSL3,BCKD-E1beta,PAHX,BUP1,DHPR,OTC,GPAM,DCI,PSAT,SSDH,ACSL5,PTE2,CYP4F11,GCSP,CTE1,HPLC2 | 31.505 | 36/385 |
| sterol biosynthesis | HMDH,Beta- ketothiolase,IDI1,FDFT1,ERG1,DHCR7,CYP51A1,NSDHL,DHC24,ACAA2 | 26.235 | 10/21 |
| metabolism | Leptin receptor,RPA3,BMAL1,CAR,V1a receptor,CYP2J2,Histone H2,Glycogen phosphorylase,RAR,TGM2,eIF4A,PLA2,SCD,ACLY,Vinculin,HMDH,HMGCS1,Galpha(q)-specific peptide GPCRs,NDPK A,RARbeta,SREBP1,Acyl-CoA synthetase,HYEP,MIF,HMGCS2,CYP4F2,HNF3-gamma,P-glycoprotein,MDR3,6PGD,G6PD,Pyruvate kinase,Carbonic anhydrase III,Fatty acid-binding protein,CYP17,CPT-1A,Tubulin beta,Beta- ketothiolase,Acetyl-CoA acyltransferase,GSTM1,INSIG1,IDI1,FDFT1,ERG1,LSS,DHCR7,Glycophorin C,CYP51A1,HNF3,ME1,HES6,SC4MOL,cytochrome P-450 reductase,GYS2,I-FABP,CAR/RXR-alpha,DDC,AFP,AMPL,CRAT,SLC34A1,ARLY,B-FABP,CPT II,S14 protein,CYP4A11,NOTCH2,APOA5,HADHB,ACSL3,AMYC,ABBP1,BCKD-E1beta,PAHX,COLQ,E-FABP,Notch,NSDHL,BUP1,DHPR,Aurora-A,OTC,MNK2(GPRK7),Guanine deaminase,Monoglyceride lipase,NAP1,AMYP,CLIM1,GPAM,ERp29,Histone H2A,TPST1,Aminopeptidase P,ACAT2,KHSRP,RDH11,PLA2G12,IBP,Histone H2B,DCI,COMT,PSAT,AFP-cyclopeptide,GSTT1,SSDH,PTPR-delta,AMYS,Nucleosome,MAO,GSTK1,ENPP3,DHC24,ACAA2,ACSL5,PTE2,PLCC,CYP4F11,GCSP,NF-I,CTE1,AAAD,HSST1,PXR,RBM3,BPHL,PMVK,EPHX2,HPLC2,HIST1H2BG,RP11217H11,eIF4A2,Cathepsin V,GLGB | 24.976 | 137/4420 |
| lipid biosynthesis | PLA2,SCD,HMDH,SREBP1,Acyl-CoA synthetase,MIF,Fatty acid-binding protein,CYP17,Beta- ketothiolase,IDI1,FDFT1,ERG1,DHCR7,CYP51A1,APOA5,ACSL3,E-FABP,NSDHL,DHC24,ACAA2,PLCC | 24.373 | 21/164 |
| cholesterol biosynthesis | IDI1,FDFT1,ERG1,DHCR7,CYP51A1,APOA5,ACSL3,E-FABP,NSDHL,DHC24,ACAA2,PLCC | 23.070 | 9/20 |
| alcohol metabolism 1 | HMDH,Galpha(q)-specific peptide GPCRs,6PGD,G6PD,Pyruvate kinase,Fatty acid-binding protein,Beta- ketothiolase,IDI1,FDFT1,ERG1,DHCR7,CYP51A1,DDC,E-FABP,NSDHL,IBP,COMT,SSDH,MAO,DHC24,ACAA2 | 8.793 | 21/221 |
| steroid metabolism | HMDH,CYP17,Beta- ketothiolase,IDI1,FDFT1,ERG1,LSS,DHCR7,CYP51A1,SC4MOL,AFP,NSDHL,AFP-cyclopeptide,DHC24,ACAA2,PXR | 18.440 | 16/128 |
| steroid biosynthesis | HMDH,CYP17,Beta- ketothiolase,IDI1,FDFT1,ERG1,DHCR7,CYP51A1,NSDHL,DHC24,ACAA2, | 18.433 | 11/53 |
| primary metabolism | RPA3,BMAL1,CAR,V1a receptor,CYP2J2,Histone H2,Glycogen phosphorylase,RAR,TGM2,eIF4A,PLA2,SCD,ACLY,Vinculin,HMDH,HMGCS1,Galpha(q)-specific peptide GPCRs,NDPK A,RARbeta,SREBP1,Acyl-CoA synthetase,MIF,CYP4F2,HNF3-gamma,P-glycoprotein,MDR3,6PGD,G6PD,Pyruvate kinase, Fatty acid-binding protein,CYP17,CPT-1A,Tubulin beta,Beta- ketothiolase,Acetyl-CoA acyltransferase,IDI1,FDFT1,ERG1,LSS,DHCR7,Glycophorin C,CYP51A1,HNF3,ME1,HES6,SC4MOL,GYS2,I-FABP,CAR/RXR-alpha,DDC,AFP,AMPL,CRAT,ARLY,B-FABP,CPT II,S14 protein,CYP4A11,NOTCH2,APOA5,HADHB,ACSL3,AMYC,ABBP1,BCKD-E1beta,PAHX,COLQ,E-FABP,Notch,NSDHL,BUP1,DHPR,Aurora-A,OTC,MNK2(GPRK7),Guanine deaminase,Monoglyceride lipase,NAP1,AMYP,CLIM1,GPAM,ERp29, Histone H2A,TPST1,Aminopeptidase P,ACAT2,KHSRP,RDH11,PLA2G12,IBP,Histone H2B,DCI,COMT,PSAT,AFP-cyclopeptide,SSDH,PTPR-delta,AMYS,Nucleosome,MAO,DHC24,ACAA2,ACSL5,PTE2,PLCC, CYP4F11,GCSP,NF-I,CTE1,AAAD,HSST1,PXR,RBM3,BPHL,PMVK,HPLC2,HIST1H2BG,RP11217H11,eIF4A2,Cathepsin V,GLGB | 18.060 | 124/4113 |
| cellular metabolism | Fatty acid-binding protein,CYP17,CPT-1A,Tubulin beta,Beta- ketothiolase,Acetyl-CoA acyltransferase,IDI1,FDFT1,ERG1,LSS,DHCR7,Glycophorin C,CYP51A1,HNF3,ME1,HES6,SC4MOL,GYS2,I-FABP,CAR/RXR-alpha,DDC,AFP,AMPL,CRAT,ARLY,B-FABP,CPT II,S14 protein,CYP4A11,NOTCH2,APOA5,HADHB,ACSL3,AMYC,ABBP1,BCKD-E1beta,PAHX,COLQ,E-FABP,Notch,NSDHL,BUP1,DHPR,Aurora-A,OTC,MNK2(GPRK7),Guanine deaminase,Monoglyceride lipase,NAP1,AMYP,CLIM1,GPAM,ERp29,Fatty acid-binding protein,CYP17,CPT-1A,Tubulin beta,Beta- ketothiolase,Acetyl-CoA acyltransferase,IDI1,FDFT1,ERG1,LSS,DHCR7,Glycophorin C,CYP51A1,HNF3,ME1,HES6,SC4MOL,GYS2,I-FABP,CAR/RXR-alpha,DDC,AFP,AMPL,CRAT,ARLY,B-FABP,CPT II,S14 protein,CYP4A11,NOTCH2,APOA5,HADHB,ACSL3,AMYC,ABBP1,BCKD-E1beta,PAHX,COLQ,E-FABP,Notch,NSDHL,BUP1,DHPR,Aurora-A,OTC,MNK2(GPRK7),Guanine deaminase,Monoglyceride lipase,NAP1,AMYP,CLIM1,GPAM,ERp29, Histone H2A,TPST1,Aminopeptidase P,ACAT2,KHSRP,RDH11,PLA2G12,IBP,Histone H2B,DCI,COMT,PSAT,AFP-cyclopeptide,SSDH,PTPR-delta,AMYS,Nucleosome,MAO,DHC24,ACAA2,ACSL5,PTE2,PLCC, CYP4F11,GCSP,NF-I,CTE1,AAAD,HSST1,PXR,RBM3,BPHL,PMVK,HPLC2,HIST1H2BG,RP11217H11,eIF4A2,Cathepsin V,GLGB | 16.975 | 124/4174 |
| sterol metabolism | Histone H2A,TPST1,Aminopeptidase P,ACAT2,KHSRP,RDH11,PLA2G12,IBP,Histone H2B,DCI,COMT,PSAT,AFP-cyclopeptide,SSDH,PTPR-delta,AMYS,Nucleosome,MAO,DHC24,ACAA2,ACSL5,PTE2,PLCC, | 14.516 | 10/61 |
| fatty acid oxidation | CYP4F11,GCSP,NF-I,CTE1,AAAD,HSST1,PXR,RBM3,BPHL,PMVK,HPLC2,HIST1H2BG,RP11217H11,eIF4A2,Cathepsin V,GLGB | 13.838 | 8/38 |
| cholesterol metabolism | HMDH,Beta- ketothiolase,IDI1,FDFT1,DHCR7,CYP51A1,NSDHL,DHC24,ACAA2 | 12.413 | 9/60 |
| biosynthesis | eIF4A,PLA2,SCD,HMDH,Galpha(q)-specific peptide GPCRs,NDPK A,SREBP1,Acyl-CoA synthetase,MIF, HMGCS2,Fatty acid-binding protein,CYP17,Beta- ketothiolase,IDI1,FDFT1,ERG1,DHCR7, Glycophorin C,CYP51A1,ME1,GYS2,ARLY,APOA5,ACSL3,E-FABP,NSDHL,BUP1,DHPR,OTC,MNK2(GPRK7), PSAT,DHC24,ACAA2,PLCC,HSST1,RBM3,RP11217H11,eIF4A2 | 12.000 | 38/853 |
| cellular response to stimulus | V1a receptor,Galpha(q)-specific peptide GPCRs,HNF3-gamma,HNF3 | 11.950 | 4/7 |
| cellular response to extracellular stimulus | V1a receptor,Galpha(q)-specific peptide GPCRs,HNF3-gamma,HNF3 | 11.950 | 4/7 |
| coenzyme metabolism | ACLY,Acyl-CoA synthetase,6PGD,G6PD,ME1,CRAT,GSTT1,GSTK1,PTE2,CTE1 | 11.733 | 10/82 |
| fatty acid beta-oxidation | Fatty acid-binding protein,CPT-1A,Acetyl-CoA acyltransferase,CPT II,HADHB,DCI | 11.724 | 6/24 |
| energy derivation by oxidation of organic compounds | Leptin receptor,V1a receptor,Glycogen phosphorylase,ACLY,Galpha(q)-specific peptide GPCRs,6PGD, G6PD,Pyruvate kinase,ME1,GYS2,CRAT,GLGB | 11.145 | 12/127 |
| neurotransmitter catabolism | COLQ,COMT,SSDH,MAO | 10.703 | 4/9 |
| nucleosome assembly | Histone H2,NAP1,Histone H2A,Histone H2B,Nucleosome,HIST1H2BG | 10.346 | 6/30 |

Gene Ontology analysis of fasting genes in the rat liver identified using EPIG.

| Process Name | Gene Name | max(-log(pValue)) | Network objects |
| --- | --- | --- | --- |
| lipid metabolism | CYP2J2,PLA2,SCD,ACLY,HMDH,HMGCS1,SREBP1,Acyl-CoA synthetase,MIF,CYP4F2,P-glycoprotein,MDR3,HSD17B2,Fatty acid-binding protein,CYP17,CPT-1A,Acetyl-CoA acyltransferase,IDI1,FDFT1,ERG1,LSS,DHCR7,FDPS,CYP51A1,SC4MOL,AFP,CRAT,B-FABP,CPT II,S14 protein, CYP4A11,HADHB,ACSL3,PAHX,E-FABP,NSDHL,GPAM,ACAT2,PLA2G12,DCI,AFP-cyclopeptide,DHC24,PTE2,CYP4F11,CTE1,PXR,PMVK,HPLC2 | 63.438 | 48/532 |
| cellular lipid metabolism | CYP2J2,PLA2,SCD,HMDH,SREBP1,Acyl-CoA synthetase,MIF,CYP4F2,HSD17B2,Fatty acid-binding protein,CYP17,CPT-1A,Acetyl-CoA acyltransferase,IDI1,FDFT1,ERG1,LSS,DHCR7,FDPS,CYP51A1SC4MOL,AFP,CRAT,B-FABP,CPT II,CYP4A11,HADHB,ACSL3,PAHX,E-FABP,NSDHL,GPAM,DCI,AFP-cyclopeptide,DHC24,PTE2,CYP4F11,CTE1,PXR,HPLC2 | 52.360 | 40/436 |
| fatty acid metabolism | CYP2J2,PLA2,SCD,Acyl-CoA synthetase,MIF,CYP4F2,Fatty acid-binding protein,CPT-1A,Acetyl-CoA acyltransferase,SC4MOL,CRAT,B-FABP,CPT II,CYP4A11,HADHB,ACSL3,PAHX,GPAM,DCI,PTE2,CYP4F11,CTE1,HPLC2 | 42.072 | 23/142 |
| carboxylic acid metabolism | CYP2J2,PLA2,SCD,ACLY,Acyl-CoA synthetase,MIF,CYP4F2,Fatty acid-binding protein,CPT-1A,Acetyl-CoA acyltransferase,SC4MOL,CRAT,ARLY,B-FABP,CPT II,CYP4A11,HADHB,ACSL3,BCKD-E1beta,PAHX,DHPR,GPAM,DCI,PSAT,PTE2,CYP4F11,CTE1,HPLC2 | 30.001 | 28/383 |
| organic acid metabolism | CYP2J2,PLA2,SCD,ACLY,Acyl-CoA synthetase,MIF,CYP4F2,Fatty acid-binding protein,CPT-1A,Acetyl-CoA acyltransferase,SC4MOL,CRAT,ARLY,B-FABP,CPT II,CYP4A11,HADHB,ACSL3,BCKD-E1beta,PAHX,DHPR,GPAM,DCI,PSAT,PTE2,CYP4F11,CTE1,HPLC2 | 29.87 | 28/385 |
| metabolism | Leptin receptor,CAR,CYP2J2,Histone H2,Glycogen phosphorylase,TGM2,PLA2,SCD,ACLY,HMDH,HMGCS1,NDPK A,SREBP1,Acyl-CoA synthetase,HYEP,MIF,HMGCS2,CYP4F2,IBP1,P-glycoprotein,MDR3,6PGD,HSD17B2,Pyruvate kinase,Carbonic anhydrase III,DBP,Fatty acid-binding protein,CYP17,CPT-1A,Tubulin beta,Acetyl-CoA acyltransferase,GSTM1,IDI1,FDFT1,ERG1,LSS,DHCR7,FDPS,CYP51A1,HES6,SC4MOL,cytochrome P-450 reductase,GYS2,CAR/RXR-alpha,DDC,AFP,AMPL,CRAT,ARLY,B-FABP,CPT II,S14 protein,CYP4A11,NOTCH2,ADH4,HADHB,ACSL3,AMYC,ABBP1BCKD-E1beta,PAHX,COLQ,E-FABP,Notch,NSDHL,DHPR,MNK2(GPRK7),AMYP,CLIM1,GPAM,ERp29,Histone H2A,TPST1,Aminopeptidase P,ACAT2,PLA2G12,IBP,Histone H2B,DCI,PSAT,AFP-cyclopeptide,GSTT1,AMYS,Nucleosome,ENPP3,DHC24,PTE2,CYP4F11,CTE1,PXR,RBM3,PMVK,EPHX2,HPLC2,HIST1H2BG,GLGB | 29.208 | 97/4420 |
| lipid biosynthesis | PLA2,SCD,HMDH,SREBP1,Acyl-CoA synthetase,MIF,HSD17B2,Fatty acid-binding protein,CYP17,IDI1,FDFT1,ERG1,DHCR7,FDPS,CYP51A1,ACSL3,E-FABP,NSDHL,DHC24 | 28.427 | 19/164 |
| sterol biosynthesis | HMDH,IDI1,FDFT1,ERG1,DHCR7,FDPS,CYP51A1,NSDHL,DHC24 | 26.595 | 9/21 |
| steroid metabolism | HMDH,HSD17B2,CYP17,IDI1,FDFT1,ERG1,LSS,DHCR7,FDPS,CYP51A1,SC4MOL,AFP,NSDHL,AFP-cyclopeptide,DHC24,PXR | 25.197 | 16/128 |
| steroid biosynthesis | HMDH,HSD17B2,CYP17,IDI1,FDFT1,ERG1,DHCR7,FDPS,CYP51A1,NSDHL,DHC24 | 23.245 | 11/53 |
| cholesterol biosynthesis | HMDH,IDI1,FDFT1,DHCR7,FDPS,CYP51A1,NSDHL,DHC24 | 23.067 | 8/20 |
| cellular metabolism | Leptin receptor,CAR,CYP2J2,Histone H2,Glycogen phosphorylase,TGM2,PLA2,SCD,ACLY,HMDH,NDPK A,SREBP1,Acyl-CoA synthetase,HYEP,MIF,HMGCS2,CYP4F2,IBP1,6PGD,HSD17B2,Pyruvate kinase,Carbonic anhydrase III,DBP,Fatty acid-binding protein,CYP17,CPT-1A,Acetyl-CoA acyltransferase,GSTM1,IDI1,FDFT1,ERG1,LSS,DHCR7,FDPS,CYP51A1,HES6,SC4MOL,cytochrome P-450 reductase,GYS2,CAR/RXR-alpha,DDC,AFP,AMPL,CRAT,ARLY,B-FABP,CPT II,S14 protein,CYP4A11,NOTCH2,ADH4,HADHB,ACSL3,ABBP1,BCKD-E1beta,PAHX,COLQ,E-FABP,Notch,NSDHL,DHPR,MNK2(GPRK7),CLIM1,GPAM,ERp29,Histone H2A,TPST1,Aminopeptidase P,IBP,Histone H2B,DCI,PSAT,AFP-cyclopeptide,GSTT1,Nucleosome,ENPP3,DHC24,PTE2,CYP4F11,CTE1,PXR,RBM3,PMVK,EPHX2,HPLC2,HIST1H2BG,GLGB | 20.533 | 88/4174 |
| primary metabolism | CAR,CYP2J2,Histone H2,Glycogen phosphorylase,TGM2,PLA2,SCD,ACLY,HMDH,HMGCS1,NDPK A,SREBP1,Acyl-CoA synthetase,MIF,CYP4F2,IBP1,P-glycoprotein,MDR3,6PGD,HSD17B2,Pyruvate kinase,DBP,Fatty acid-binding protein,CYP17,CPT-1A,Tubulin beta,Acetyl-CoA acyltransferase,IDI1,FDFT1,ERG1,LSS,DHCR7,FDPS,CYP51A1,HES6,SC4MOL,GYS2,CAR/RXR-alpha,DDC,AFP,AMPL,CRAT,ARLY,B-FABP,CPT II,S14 protein,CYP4A11,NOTCH2,HADHB,ACSL3,AMYC,ABBP1,BCKD-E1beta,PAHX,COLQ,E-FABP,Notch,NSDHL,DHPR,MNK2(GPRK7),AMYP,CLIM1,GPAM,ERp29,Histone H2A,TPST1,Aminopeptidase P,ACAT2,PLA2G12,IBP,Histone H2B,DCI,PSAT,AFP-cyclopeptide,AMYS,Nucleosome,DHC24,PTE2,CYP4F11,CTE1,PXR,RBM3,PMVK,HPLC2,HIST1H2BG,GLGB | 20.230 | 87/4113 |
| alcohol metabolism | HMDH,IBP1,6PGD,Pyruvate kinase,Fatty acid-binding protein,IDI1,FDFT1,ERG1,DHCR7,FDPS,CYP51A1,DDC,ADH4,E-FABP,NSDHL,IBP,DHC24 | 19.012 | 17/221 |
| fatty acid oxidation | Fatty acid-binding protein,CPT-1A,Acetyl-CoA acyltransferase,CPT II,HADHB,PAHX,DCI,HPLC2 | 17.307 | 8/38 |
| sterol metabolism | HMDH,IDI1,FDFT1,ERG1,DHCR7,FDPS,CYP51A1,NSDHL,DHC24 | 16.061 | 9/61 |
| fatty acid beta-oxidation | Fatty acid-binding protein,CPT-1A,Acetyl-CoA acyltransferase,CPT II,HADHB,DCI | 14.342 | 6/24 |
| cholesterol metabolism | HMDH,IDI1,FDFT1,DHCR7,FDPS,CYP51A1,NSDHL,DHC24 | 13.597 | 8/60 |
| biosynthesis | PLA2,SCD,HMDH,NDPK A,SREBP1,Acyl-CoA synthetase,MIF,HMGCS2,HSD17B2,Fatty acid-binding protein,CYP17,IDI1,FDFT1,ERG1,DHCR7,FDPS,CYP51A1,GYS2,ARLY,ACSL3,E-FABP,NSDHL,DHPR,MNK2(GPRK7),PSAT,DHC24,RBM3 | 10.893 | 27/853 |
| acyl-CoA metabolism | Acyl-CoA synthetase,CRAT,PTE2,CTE1 | 10.163 | 4/15 |
| nucleosome assembly | Histone H2,Histone H2A,Histone H2B,Nucleosome,HIST1H2BG | 10.009 | 5/30 |
| icosanoid metabolism | CYP2J2,PLA2,MIF,CYP4F2,CYP4F11 | 9.239 | 5/35 |
| coenzyme metabolism | ACLY,Acyl-CoA synthetase,6PGD,CRAT,GSTT1,PTE2,CTE1 | 9.087 | 7/82 |
| chromatin assembly | Histone H2,Histone H2A,Histone H2B,Nucleosome,HIST1H2BG | 8.587 | 5/40 |
| recombinational repair | Histone H2,Histone H2A,Nucleosome | 8.219 | 3/10 |
