# Supplementary material for: Sources of variation in baseline gene expression levels from toxicogenomics study control animals across multiple laboratories
Source: BMC Genomics. 2008 Jun 12;9:285. doi: 10.1186/1471-2164-9-285 (PMC2453529; doi:10.1186/1471-2164-9-285)
Supplement: Additional file 14 — Comparison of gender-specific and fasting genes to genes with the highest baseline variability. This table contains a comparison of probe sets in common between a list of RAE230A probe sets ranked in order of highest to lowest baseline variability and lists of probe sets regulated by gender or fasting. [file 1471-2164-9-285-S14.doc]

| # Genes queried out of 15,924 (% of total) | % of Fasting genes identified | % of Liver gender-specific genes identified | % of Kidney gender-specific genes identified |
| --- | --- | --- | --- |
| 100 (0.6) | 3.0 | 9.8 | 3.3 |
| 500 (3.1) | 34.1 | 34.0 | 14.8 |
| 1000 (6.3) | 56.7 | 58.9 | 22.0 |
| 2000 (12.6) | 80.2 | 81.5 | 33.8 |
| 5000 (31.4) | 98.2 | 97.4 | 55.7 |
