# Supplementary material for: Sources of variation in baseline gene expression levels from toxicogenomics study control animals across multiple laboratories
Source: BMC Genomics. 2008 Jun 12;9:285. doi: 10.1186/1471-2164-9-285 (PMC2453529; doi:10.1186/1471-2164-9-285)
Supplement: Additional file 15 — Primer sequences for real-time RT-PCR analysis of gender-selective genes. This file lists the gene symbol, GenBank number, and forward and reverse primer sequences for the 34 test genes and 2 control genes used to verify gender-selective expression. [file 1471-2164-9-285-S15.doc]

Primer sequences for real-time RT-PCR analysis of selected genes

|  | GenBank |  |  |
| --- | --- | --- | --- |
| Gene | Number | Forward | Reverse |
| 18S | X56974 | CGAACGTCTGCCCTATCAACTT | CCGGAATCGAACCCTGATT |
| A1bg | AJ302031 | CCGTAGAGCTGATGTGGAGTGA | AGGCTCCAGGTTGTGACTTGA |
| Acox2 | X95189 | CATGCACGCCTTCATTGTG | GGGCCTATGTCCCCAACTGT |
| Alcam | NM_031753 | CGGTGATACCCTGCCTGTGT | GATCGGAGCCTGATGTTATCCT |
| Afm | X76456 | CATGGAAGAACTGGTCTCCCTTAG | ACAGGCGAACTCGTCACTGA |
| Akr1b7 | NM_053781 | GCAGCCATTGATGCTGGATA | TGGCTTCTCCCACCTCACTCT |
| Akr1c12 | XM_341549 | GCTGAGCCGTCCAGATTGAT | TGTGTGGACAGTGATGCTGATG |
| Akr7a3 | NM_013215 | CCGCTTCTTTGGGAATCCAT | GGCGATGCCATTGAAGTGTT |
| Angpt2 | BI275292 | CTTGTGACAGAGTCCGAATGCA | CGGGTCTGGAGAAATACCTATGAG |
| beta-actin | V01217 | TCCTCCTGAGCGCAAGTACTCT | GCTCAGTAACAGTCCGCCTAGAA |
| CD36 | NM_031561 | GGCTAAATGAGACTGGGACCAT | CCAGGCCCAGGAGCTTTATT |
| Ces1 | NM_031565 | TGGGTCCAGGACAACATTGA | GACACTTTCACCTCCTGCTGACT |
| Ces3 | L46791 | CGGAATGGGAGCCCTAATG | CTGGGCTGCCTGAGTTGAG |
| Cited2 | AI013390 | GAGACGGCAGTTTGTGCAGTAA | CTGCGCACTGCATTCAGATT |
| Cldn3 | NM_031700 | GACCACCCCACCTTCCAGAT | GCCAGGCTGTCTGTCCTCTT |
| Crot | J02844 | TGGAAGAGTGGTGGCTCAATG | TGGAAGAGTGGTGGCTCAATG |
| Cyp2c40 | NM_031572 | GCAGAGTGGCTAGTGATGGAACT | TGCAGGGAGCACATCCTATG |
| Cyp3a11 | U09742 | AAACCAC CAGCAGCACACTCT | CAGGGCCCCATCGATCTC |
| Cyp4b1 | M29853 | CCGAAGGCTGCAGATGTGTA | CATTTTGGCCCATCCAGAAC |
| Dnaja4 | AI104324 | ACCCACCTTCTAGGCCTTTGTT | GGCTTTGCCATGGTTTGTTC |
| Eif2s3x | AI598546 | AACCTCCATGTCACCCACAGA | TTCCACCCATGACCCTTCAA |
| Ela1 | NM_012552 | TCCCCAAGATGGTGCTTAGC | TGCCGCTGAGCGATAACC |
| Fdx1 | NM_017126 | CTGGGCTGTCAAGTTTGTCTGA | GGACATCTGCCACTGCTTCA |
| Hdac2 | AA892297 | CCTACGACCTCCTTCACCTTCA | GATGGAGAAGACCCTGACAAGAGA |
| Jak2 | NM_031514 | AACTGCGGATGCACACCATT | TGGGCCGTGACAGTTGCT |
| Oat | NM_022521 | GAGCCCATCCAGGGTGAAG | GTGCCGGGTGCAAAGTTC |
| Prep | NM_031324 | CCCAATTACCGGCTGATCAA | CGTGCTCCGGAACGAGAA |
| Prlr | NM_012630 | TGCAAGCCAGACCATGGATAC | CACGGTTGTGTCCTTCAAGGT |
| Prom1 | NM_021751 | CAGACCCAGCCAGCAAGATC | TGGGAGCTGACTTCCGTTGT |
| Rgn | NM_031546 | ATCGAGTGCAGCGAGTTGGT | GTGGCAACATAGCCTCCTGACT |
| Slc22a2 | NM_031584 | CTGTTCTCTACCAGGGCCTCAT | GGGCGGAGTAGAAGAAATCCA |
| Slc22a5 | NM_019269 | GCGCCTTCCACTATCTTCGA | TTCGTGTTCGGACCAGATCA |
| Slc22a7 | NM_053537 | GCGTTGCAAAGACCCTCATAC | CCATCATGCAGCACAGTGAGA |
| Spink1 | M27882 | GCTTTGGCCCTGCTCAGTT | GGGTCATAAATCCTGGGACATC |
| Stmn1 | NM_017166 | GAAGCGGGAGCATGAAAAAG | CTCCTCTGCCATTTTGCTGAAG |
| Yc2 | AA945082 | GGATACTCCAGCGTCTAAGAAGCT | GCTGGGATGCCATCTAGTTGA |
